# Metabolic engineering of *Vibrio natriegens* for the efficient biosynthesis of ergothioneine from sucrose using non-sterile fed-batch fermentation

**DOI:** 10.1016/j.synbio.2026.04.041

**Published:** 2026-06-12

**Authors:** Xinhui Liang, Yue Wang, Yifei Lv, Chaoyong Huang, Zhenbang Huang, Jinfeng Wei, Shijie Jiang, Zhiyang Dong, Zhengfu Zhou, Min Lin

**Affiliations:** a Engineering Research Center of Biomass Materials, Ministry of Education, College of Life Sciences and Agri-forestry, Southwest University of Science and Technology, Mianyang, Sichuan, 621010, PR China; b Food Laboratory of Zhongyuan, College of Agriculture, Henan University, Kaifeng, 475001, PR China; c Shenzhen Siyomicro Bio-Tech Co., Ltd. Shenzhen, Guangdong, 518057, PR China; d Key Laboratory of Agricultural Microbiome (MARA), Biotechnology Research Institute, Chinese Academy of Agricultural Sciences, Beijing, 100081, PR China

**Keywords:** *Vibrio natriegens*, Ergothioneine, Metabolic engineering, CRISPR-Cas9, Non-sterile fermentation

## Abstract

Fast-growing *Vibrio natriegens* is now recognized as a next-generation chassis for synthetic biology and biotechnology; however, its low transformation efficiency, limited gene editing methods and high fermentation cost are still the main challenges hampering its industrial application. In this study, we established an efficient electroporation transformation and dual-plasmid CRISPR-Cas9 editing system in *V. natriegens*. Subsequently, the heterologous ergothioneine biosynthetic pathway involving the combination of the superstrong P_L_*lacO1* promoter and weak RBS7 was constructed in *V. natriegens*. Multiple genes encoding genes involved in byproduct formation and adenosine triphosphate (ATP) degradation were consecutively deleted, while several key genes involved in the S-adenosylmethionine (SAM) cycle and the ATP synthesis pathway were overexpressed to increase ergothioneine production. Finally, fed-batch fermentation was performed using low-cost sucrose as the sole carbon source under high-salinity, non-sterile conditions, resulting in an ergothioneine titer of 1.2 g/L in a 2-L bioreactor. This study not only provides the first successful example of the ergothioneine biosynthesis with engineered *V. natriegens* strains but also establishes an efficient and economic platform in which *V. natriegens* is used to produce other high-value compounds.

## 1 Introduction

Ergothioneine (EGT), which was first isolated from *Claviceps purpurea* more than a century ago, is a rare natural thiohistidine derivative with potent antioxidant properties and diverse biological functions [1,2]. Ergothioneine biosynthesis occurs specifically in bacteria and fungi but not in plants or animals [[3], [4], [5], [6], [7]]. Five bacterial genes (*egtABCDE*) in *Mycobacterium smegmatis* were confirmed to be involved in ergothioneine biosynthesis [8,9]. In the bacterial biosynthetic pathway, EgtD catalyzes the conversion of l-histidine (L-His) to l-histidine betaine (HER) via its triple methylation by S-adenosylmethionine (SAM). EgtB subsequently catalyzes O_2_-dependent C–S bond formation between γ-glutamyl cysteine (γGC), which is supplied by EgtA, and HER to yield γ-l-glutamyl-S-(hercyn-2-yl)-l-cysteine sulfoxide (γGC-HER). Subsequently, the aminohydrolase EgtC partially removes l-glutamic acid (L-Glu) to generate cysteine sulfoxide (Cys-HER). In addition, one fungal gene encoding the ergothioneine biosynthetic protein NcEgt1 and the ergothioneine biosynthetic C–S lyase NcEgt2 were first identified in *Neurospora crassa* [10]. *Trichoderma reesei* also possesses key genes encoding the SAM-dependent histidine methyltransferase TrEgt1 and the PLP-mediated C–S lyase TrEgt2, which are involved in ergothioneine biosynthesis [[11], [12], [13], [14]]. Under Fe^2+^/O_2_ conditions, TrEgt1 catalyzes the condensation of HER and cysteine to Cys-HER, and TrEgt2 then converts Cys-HER to ergothioneine [15]. Although many microorganisms can synthesize ergothioneine, its natural production by wild-type strains falls significantly short of industrial requirements.

With the characterization of ergothioneine biosynthetic pathways in bacteria and fungi, *de novo* ergothioneine biosynthesis by engineered microorganisms, such as *Escherichia coli*, *Saccharomyces cerevisiae*, and *Corynebacterium glutamicum*, has gradually become the mainstream method [8,9]. Due to output and cost constraints, only a few engineered strains can be practically applied to the industrial-scale production of ergothioneine [[3], [4], [5], [6], [7]]. Thus, the discovery and remodeling of more suitable microorganisms as microbial platforms are urgently needed to develop metabolically engineered strains with fast cell proliferation, high stress tolerance, a broad substrate spectrum, and low byproduct formation.

*Vibrio natriegens*, a fast-growing marine bacterium with broad substrate utilization and superior stress tolerance, has emerged as a promising next-generation industrial chassis for synthetic biology applications [16,17]. A key attribute underpinning this potential is its characteristics of low nutritional requirements and robust growth. It can double in less than 10 min, and its growth rate in nutrient-limited media still exceeds that of *E. coli* by more than twofold [18,19]. In addition, *V. natriegens* is a marine bacterium that is resistant to high salt concentrations up to 30 g L^−1^, with an optimum concentration of 15 g L^−1^ and its fast growth could help maintain axenic fermentation conditions. Similarly, *V. natriegens* has a substrate uptake rate that is more than twice that of *E. coli*, which directly contributes to its higher potential productivity [18,20]. In addition to these growth-related advantages, *V. natriegens* offers several practical benefits for bioprocessing. Its inherent halotolerance facilitates axenic or even open fermentation strategies, thereby significantly reducing sterilization costs [21,22]. Furthermore, it is a nonpathogenic organism classified under BSL-1, with no known cases of human infection, ensuring a high safety profile for industrial use [23]. From a metabolic perspective, *V. natriegens* possesses a broad substrate spectrum and is capable of utilizing diverse carbon sources, such as glycerol, arabinose, galactose, sucrose, and glucose, highlighting its versatility in feedstock utilization [18,24]. Critically, the development of a comprehensive suite of genetic tools and omics resources has rendered *V. natriegens* highly amenable to metabolic engineering [18,20,[25], [26], [27], [28], [29], [30], [31], [32], [33], [34], [35], [36], [37], [38]]. Inspired by these inherent *V. natriegens* characteristics, engineered *V*. *natriegens* has shown considerable potential for the industrial-scale production of a diverse array of compounds, frequently surpassing traditional hosts such as *E*. *coli* in terms of volumetric productivity [39]. Several genome engineering tools, including multiplex genome editing by natural transformation, have been developed for *V. natriegens* to fully harness its potential, and are indispensable for the generation of strains tailored for specific applications. Successful case studies include the synthesis of amino acids [18,40], polyols [21,41,42], pigments such as melanin [43], the blue pigment indigoidine [44], β-carotene and violacein [24], as well as organic acids such as succinate [[3], [26]]-hydroxypropionate [45]. Notably, metabolic engineering efforts have led to substantial improvements in yield, with reported increases in productivity of about 3-fold for compounds such as 2,3-butanediol [21,41] and a remarkable 13-fold increase for alanine [18]. This broad spectrum of successful applications not only underscores the versatility of *V. natriegens* as a microbial platform but also strongly indicates that the metabolic engineering strategies validated for these products are potentially transferable to the biosynthesis of other high-value compounds.

Despite this genetic potential, a comprehensive understanding of how *V. natriegens* metabolizes sucrose is still lacking. Little is known about the enzymes, regulatory nodes, and carbon flux distribution supporting low nutritional requirements and robust growth in this organism or how its metabolism may be optimized for the sucrose-based bioproduction of ergothioneine. These knowledge gaps hinder rational pathway engineering and process optimization for production of various value-added compounds in *V. natriegens*. Additionally, the tremendous potential of gene editing systems in *V. natriegens* is still hampered because of transformation efficiencies that are much lower than those observed for highly engineered *E. coli* strains [36]. Although functional genes involved in the synthesis of ergothioneine have not been identified in the *V. natriegens* genome, we detected low concentrations of ergothioneine in the fermentation broth in a preliminary study, suggesting the presence of a putative endogenous biosynthetic pathway that differs from those reported in *M. smegmatis*, *T. reesei*, and *N. crassa.* However, successful examples of industrial production of ergothioneine in *V. natriegens* have not yet been described.

In this study, we established an efficient electroporation transformation and dual-plasmid CRISPR-Cas9 editing system in *V. natriegens*. Using these methods, various pathways, including heterologous ergothioneine synthesis, byproduct synthesis pathways, and the SAM or adenosine triphosphate (ATP) supply, were investigated in *V. natriegens*, and a set of metabolically engineered strains was constructed for efficient ergothioneine production. Fed-batch fermentation was performed using low-cost sucrose as the sole carbon source under high-salinity, non-sterile conditions, resulting in an ergothioneine titer of 1.2 g/L and a productivity of 50 mg/L/h.

## 2 Materials and methods

### 2.1 Strains and plasmids

The strains and plasmids used in this study are shown in Table S1. *E. coli* DH5α was routinely employed as the host strain for plasmid construction and DNA manipulation. All genetic modules for ergothioneine production were assembled in the expression vector pCOlE1 using *V. natriegens* ATCC 14048 (purchased from Shenzhen EaseBio Technology Co., Ltd. (Shenzhen, China; Cat. No. CICC10908)) as the parental strain.

### 2.2 Medium and culture conditions

Luria–Bertani (LB) medium (10 g/L tryptone, 5 g/L yeast extract, and 10 g/L NaCl) was used to grow *E. coli* strains. LB3 medium consisting of 10 g/L tryptone, 5 g/L yeast extract, and 30 g/L NaCl was used for the cultivation of the *V. natriegens* strains. The *E. coli* or *V. natriegens* strains were initially grown with shaking at 220 rpm at either 37 °C or 30 °C.

Modified minimal medium M9 (Na_2_HPO_4_ 6.75 g/L, KH_2_PO_4_ 3 g/L, NH_4_Cl 1 g/L, NaCl 32 g/L, sucrose 32 g/L, yeast extract 5 g/L, and 1 mL of a trace element solution composed of FeCl_3_·6H_2_O 2.4 g/L, ZnCl_2_ 0.3 g/L, Na_2_MoO_4_·2H_2_O 0.3 g/L, MnCl_2_·4H_2_O 0.5 g/L, CoCl_2_·6H_2_O 0.3 g/L, CuCl_2_·2H_2_O 0.15 g/L, and H_3_BO_3_ 0.08 g/L, pH 7.0) was used for *V. natriegens* growth in shake flasks. Where necessary, 150 μg/mL kanamycin (Kan), 2 μg/mL chloramphenicol (Cm), 100 μg/mL ampicillin (Amp), 20 g/L histidine, 20 g/L cysteine, 40 g/L methionine, and 200 g/L arabinose, 23.8 g/L isopropyl β-d-1-thiogalactopyranoside (IPTG) were added.

### 2.3 Preparation of electrocompetent cells and electroporation

A single colony of *V. natriegens* was inoculated into 2 mL of LB3 and incubated at 37 °C and 220 rpm overnight. A 100-μL aliquot of the seed culture was transferred to 100 mL of fresh LB3 and grown at 30 °C and 220 rpm to an OD_600_ ≈ 0.5. The cells were chilled on ice for 30 min and then harvested by centrifugation (4 °C, 5000 rpm, 10 min). The pellet was gently resuspended in 10 mL of ice-cold 1 M sorbitol, recentrifuged under the same conditions, and washed three additional times (four washes total) with 10 mL of ice-cold 1 M sorbitol. The final pellet was resuspended in 500 μL of ice-cold 1 M sorbitol, dispensed as 100 μL aliquots, and stored at −80 °C if not used immediately. For electroporation, ∼200 ng of pUC19 plasmid DNA was added to 100 μL of competent cells, mixed gently, and incubated on ice for 30 min. The mixture was transferred to a prechilled 1-mm cuvette and pulsed at 900 V, 25 μF, and 200 Ω. Immediately, 1 mL of LB3 was added, and the cells were transferred to a 1.5 mL tube and allowed to recover at 37 °C and 220 rpm for 45 min. After brief centrifugation, the cells were plated on LB3 agar containing the appropriate antibiotic and incubated at 37 °C overnight.

### 2.4 Establishment of the dual-plasmid CRISPR-Cas9 editing system in *V. natriegens*

In this study, a dual-plasmid CRISPR-Cas9 genome editing system comprising a helper vector, pColE1-Cas9, and a targeting repair vector, p15A-sgRNA, was developed in *V. natriegens*.

First, the helper vector pColE1-Cas9 was constructed by integrating an l-arabinose-inducible cas9 nuclease, a P_L_*lacO1*-driven λ-Red recombinase system (*exo*, *bet*, and *gam*), and a *sacB* counterselection marker into a medium-copy backbone. Complementarily, the targeting repair vector p15A-sgRNA was engineered to carry an inducible sgRNA expression cassette and a donor DNA template. This template consisted of ∼500 bp homology arms flanking an endogenous *upp* counterselection marker, which was assembled with the vector backbone via Gibson assembly to facilitate homologous recombination. Next, sgRNAs targeting the gene of interest were designed using the CHOPCHOP online tool (https://chopchop.cbu.uib.no), with candidates prioritized based on high predicted cleavage efficiency and specificity. Following sequence verification, the pColE1-Cas9 and p15A-sgRNA plasmids were introduced into *V. natriegens* via electroporation. Genome editing was executed through the sequential induction of λ-Red recombinase and Cas9 expression. Successful transformants were further processed for the selection and genomic verification of the desired modifications.

Gene deletion or integration experiments in *V. natriegens* were performed using the dual-plasmid CRISPR-Cas9 genome editing system. Briefly, strains harboring the pColE1-Cas9 and p15A-sgRNA plasmids were cultured overnight in LB3 medium supplemented with 150 μg/mL Kan and 100 μg/mL Amp at 37 °C with shaking at 220 rpm. A 400 μL aliquot of the overnight culture was centrifuged at 12,000 rpm for 1 min, after which the supernatant was discarded. The pellet was resuspended in 2 mL of LB3 medium containing 150 μg/mL Kan and 100 μg/mL Amp, followed by an incubation at 37 °C with shaking at 220 rpm for 30 min. Subsequently, 20 μL of IPTG was added to induce the accumulation of *λ-Red* recombinase, and the culture was further incubated under the same conditions for another 30 min. Then, 40 μL of 200 g/L Ara was added, and the culture was incubated for an additional 3 h. Aliquots of 1 μL and 0.1 μL of the treated culture were spread-plated on solid LB3 agar plates supplemented with 150 μg/mL Kan, 100 μg/mL Amp, and 200 g/L Ara. Correct deletion or integration was verified by colony PCR.

Forty-seven colonies were randomly selected from the recovery plates to quantify the editing efficiency of each of these deletions. Primers complementary to sequences upstream and downstream of the homology arms on the genome were used to verify accuracy and eliminate interference from residual plasmid DNA (Table S2). The PCR products were subsequently sequenced to verify the positive clones, and wild-type strains were used as controls. Editing efficiency was calculated as the ratio of positive colonies that displayed the expected band shift (reflecting target gene deletion) to the total number of colonies screened. Five representative positive clones were randomly selected, and the PCR-amplified fragments were subjected to DNA sequencing to further confirm editing fidelity. The obtained sequences were aligned with the reference genome to verify the accuracy of the junction regions.

### 2.5 Constructions of mutants and engineered strains

The bacterial strains, plasmids, and primers used, as well as the mutant and engineered strains constructed in this study, are listed in Table S1 and Table S2. Gene knockout in *V. natriegens* strain ATCC 14048 was performed using a CRISPR-Cas9 editing protocol established by our group, as shown in Table S1.

Here, the process of *dns* deletion is presented as an example. First, the CHOPCHOP (https://chopchop.cbu.uib.no) online tool was used to select *V. natriegens* as the host organism and to screen for sgRNAs targeting the *dns* gene. An sgRNA with high predicted knockout efficiency and no dimerization risk was chosen. Based on the genomic location of the selected sgRNA, two homology arms (HA1 and HA2), each at least 500 bp in length, were designed upstream and downstream of the target site to facilitate clear differentiation during subsequent verification. The primers listed in Table S2 were used to amplify HA1 and HA2 from the genome. These fragments were subsequently joined by overlapping PCR to generate the homologous recombination fragment. The fragment was subsequently cloned and inserted into the editing vector via Gibson assembly, yielding the recombinant plasmid p15A-sgRNA-Amp. After verification, the resulting plasmid was electroporated into ATCC 14048 competent cells already harboring the pColE1-Cas9-Kan plasmid, enabling CRISPR-Cas9-mediated knockout of the *dns* gene. Other mutants of the *V. natriegens* strain were generated using the same procedure.

The overexpression vectors were constructed with the plasmid pColE1 (Invitrogen, USA) as the backbone, which includes the pColE1 origin and the P_L_*lacO1* promoter induced by IPTG and the rrnB terminator. First, the primer pair F8A/R8A, which contained the P_L_*lacO1* promoter sequence, was used to amplify the 7A plasmid. Subsequently, the 8A plasmid was constructed by Gibson assembly (Vazyme Biotech Co., China) using this linearized product as the backbone and then transformed into *E. coli* DH5α for cloning and sequencing. The resulting 8A plasmid, in which tregt1 or tregt2 was under the control of the P_L_*lacO1* promoter and *rrnB* terminator, was used to transform V.nEgt01 to obtain the recombinant strain V.nEgt01 (8A). We adopted the same strategy to construct the expression plasmids 8B and 8C. We adopted the same strategy to construct the expression plasmids 8B and 8C (Table S1) . The resulting plasmids were subsequently transformed into V. natriegens ATCC 14048 to obtain the recombinant strains V. nEgt01(8B) and V. nEgt01(8C).

In addition, a set of target genes used for the construction of engineered strains included the SAM cycle genes *mtn* encoding S-adenosylhomocysteine nucleosidase (GenBank accession: WP_021463489.1), *metE* encoding cobalamin-independent methionine synthase (GenBank accession: WP_021460893.1), *metF* encoding 5,10-methylenetetrahydrofolate reductase (GenBank accession: WP_021461481.1), and *luxS* encoding S-ribosylhomocysteine lyase (GenBank accession: WP_005238123.1); and the ATP supply-related genes *prs* encoding phosphoribosyl pyrophosphate synthase (GenBank accession: NP_388873.1), *adk* encoding adenylate kinase (GenBank accession: NP_414527.1) and *apt* encoding adenine phosphoribosyltransferase (GenBank accession: NP_418291.1).

### 2.6 Selection and functional testing of gene expression elements

The Universal Bacterial Expression Resource (UBER) system, which was originally designed to provide the same relative levels of protein expression across a range of bacterial species [46], was adopted to test the promoter and ribosome binding site (RBS), with slight modifications in *V. natriegens*. Sixteen promoters and 12 RBS sequences were selected from the iGEM registry (https://www.igem.org) and designed to adjust the level of the final output of the system and drive sfGFP expression. Each promoter–RBS pair was subsequently assembled into a vector carrying a superfolder green fluorescent protein (sfGFP) reporter gene for individual analysis. The expression level of each construct was quantified by measuring both the OD_600_ and sfGFP fluorescence. Briefly, the constructed vectors were first inoculated into 2 mL of LB3 medium and cultured overnight. Afterward, 1% (v/v) of this overnight seed culture was transferred into 2 mL of fresh LB3 medium and cultivated continuously for 8 h. The samples were then analyzed using a spectrophotometer to measure the optical density at 600 nm (OD_600_) and the fluorescence intensity (with excitation at 480 nm and emission at 520 nm). The promoter strength was assessed using the medium-strength RBS (B0034) as a standardized translational control to enable cross-comparisons. Conversely, RBS strength was evaluated by pairing each RBS with a constant, strong promoter, J23101.

### 2.7 Non-sterile fed-batch fermentations

Fed-batch fermentations of engineered *V. natriegens* were performed in a 2 L bioreactor with a working volume of 750 mL under high-salinity, non-sterile conditions. Briefly, the seed culture was inoculated at 1% (v/v) into non-sterile M9 medium, in which the NaCl concentration was adjusted to 40 g/L. The cultivation conditions were as follows: temperature, 37 °C; dissolved oxygen, 100% (via agitation and aeration control); and agitation speed, 300 rpm. The pH was automatically controlled at 7.0 by adding an ammonia solution. When a noticeable increase in the dissolved oxygen content was observed, a 500 g/L sucrose feed solution was added. After 24 h of non-sterile fed-batch fermentation, the fermentation broth was centrifuged at 13,000 rpm for 3 min. Subsequently, the supernatant was diluted to an appropriate concentration and then filtered through a 0.22 μm aqueous phase filter membrane. Fermentation broths were taken at regular intervals to determine the optical density (OD), and then the cell debris and the supernatant obtained after centrifugation and filtration were used to measure dry cell weight (DCW) and ergothioneine content. To prove that the biomass at the end of fermentation is predominantly *V. natriegens* rather than other salt-tolerant contaminants during the non-sterile fermentation process, we took three samples of the fermentation culture at 12-h and 24-h intervals, respectively. The bacterial cultures were streaked on LB plates and then placed in a 37 °C incubator for 18 h. Individual colonies were imaged on an Epson Perfection V850 Pro photo scanner, and images were captured using an AxioCam506 color camera. This possibility was further checked by colony PCR and 16S rDNA sequencing of 10 single colonies randomly picked from each plate. The regions of the bacterial 16S rRNA gene were amplified using the universal primers 515F (5′-GGGGCTCAACCTCGGAATAG-3′) and 806R (5′-CTTCGCCACCGGTATTCCTT-3′).

### 2.8 Protein expression and identification

The method used here was modified slightly from the procedure described by Ruan et al. [47]. ATCC14048 and the engineered strains derived from it were cultivated in LB3 medium supplemented with ampicillin at 37 °C on a rotary shaker (220 rpm) until the optical density of the cultures at 600 nm reached 0.5. Expression was induced by the addition of isopropyl β-d-1-thiogalactopyranoside (IPTG) at a final concentration of 2% (w/v). After 12 h of induction at 37 °C and 220 rpm, the cells were collected by centrifugation and resuspended in 1 M sorbitol buffer (pH 7.0). The cell suspension was sonicated and centrifuged (12,000×*g*, 5 min). The supernatant was used for SDS‒PAGE analysis.

### 2.9 HPLC analysis of ergothioneine

An HPLC system equipped with an ultraviolet (UV) detector and a C18-T column (Agilent ZORBAX Eclipse Plus, 250 mm × 4.6 mm, 5 μm) was used for ergothioneine detection [11]. The chromatographic conditions were as follows: mobile phase consisting of a heptafluorobutyric acid (HFBA)-containing aqueous solution and methanol at a volume ratio of 7:93 and a flow rate of 1 mL/min. Ergothioneine standards (Stds) were dissolved in 70% acetonitrile. The produced ergothioneine was detected at 254 nm by comparison with the retention time of the analytical ergothioneine standard (Sigma). The concentration was quantified by dividing the slope of the standard curve by the peak area. By using the above method, the contents of intracellular and extracellular ergothioneine were determined. Unless otherwise specified, the production of ergothioneine mentioned in this paper refers to the amount of extracellular ergothioneine.

### 2.10 Quantification and statistical analysis

Unless stated otherwise, the experiments were performed three times with similar results. Each bar on the graph represents the mean of biological replicates, and the error bars indicate the SEMs (standard errors of the means), as mentioned in the figure legends for each experiment. For all the assays, n represents the number of biological replicates. Statistical analyses were performed using GraphPad Prism 8.0. Two-tailed unpaired Student's t tests with 95% confidence intervals were used to evaluate the differences between two groups. For comparisons of more than two groups, one-way ANOVA was used. A probability value of P ≤ 0.05 was considered significant. The data are shown as the average values ± SEMs. ∗∗∗∗p ≤ 0.0001; ∗∗∗p ≤ 0.001; ∗∗p ≤ 0.01; ∗p ≤ 0.05; ns: not significant.

## 3 Results

### 3.1 Establishment and optimization of the dual-plasmid CRISPR-Cas9 editing system in *V. natriegens*

**Fig. 1:**
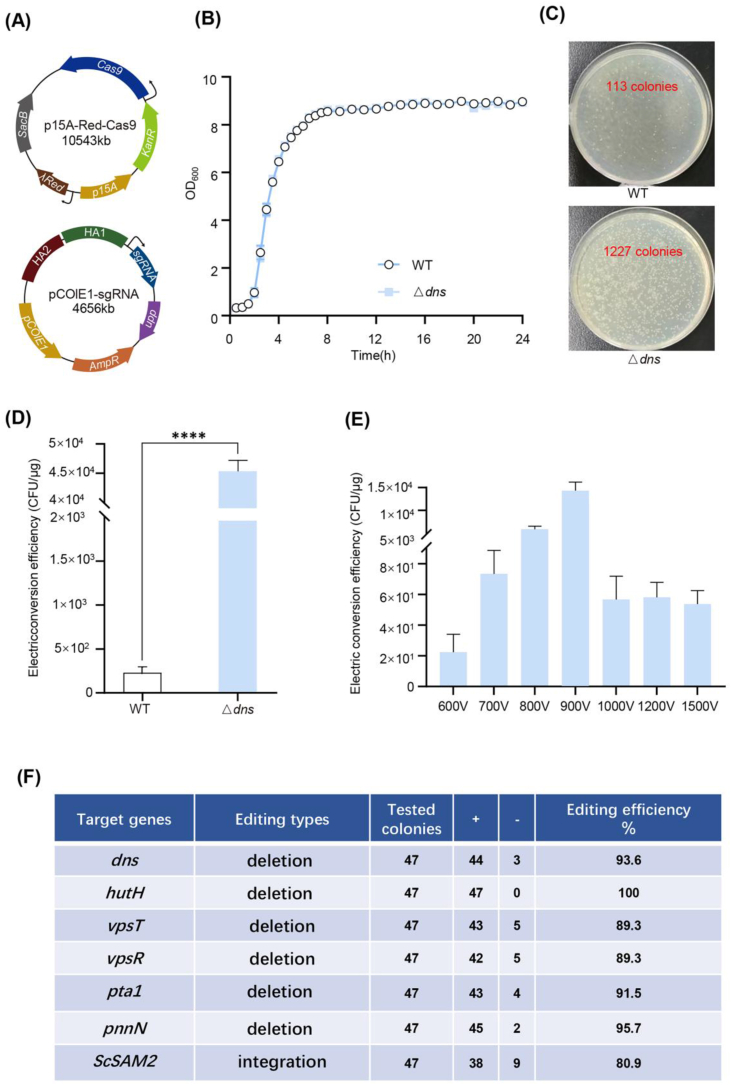
**Establishment and optimization of the CRISPR-Cas9 editing system in *V. natriegens*.** (A) Schematic representation of the dual-plasmid CRISPR-Cas9 editing system comprising a helper vector, pColE1-Cas9, and a targeting repair vector, p15A-sgRNA. More details regarding the assembly of pColE1-Cas9 and p15A-sgRNA are provided in the Materials and methods section. (B) Growth of the *V. natriegens* wild type strain and the *dns*-deleted strain in LB3 medium. (C) Representative images of plates comparing the yield of pUC19 plasmid electrotransformants of the wild type and *dns* mutant strains. (D) Electroporation transformation efficiency of the wild type and *dns* mutant strains. (E) Effect of different pulse voltages on the electroporation transformation efficiency of the *dns* mutant. (F) Editing efficiency of the target genes tested in this study. Table providing information about deleted or integrated sequences. +, positive colonies; -, negative colonies.

The CRISPR/Cas9 gene-editing system has emerged as a revolutionary tool in microbial genome engineering and has significantly accelerated the efficient and precise construction of microbial strains and the optimization of metabolic pathways [48]. In this study, we established a dual-plasmid CRISPR-Cas9 editing system in *V. natriegens*. As shown in Fig. 1A, this system contains the Cas9 expression cassette located on a p15A-based plasmid (p15A-Cas9) and the sgRNA expression cassette and homology arms (HA1 and HA2) assembled on a pCOlE1-based plasmid (pCOlE1-sgRNA). The tremendous potential of gene editing systems in *V. natriegens* is still limited because of very low transformation efficiencies. Therefore, we chose the *dns* gene, encoding an extracellular Dns nuclease, as the first target to demonstrate gene deletion by the dual-plasmid CRISPR-Cas9 editing system in *V. natriegens* since this gene is known to degrade plasmid DNA, thereby compromising transformation efficiency [29]. The growth rate of the *dns* mutant was similar to that of the WT strain in LB3 medium (Fig. 1B), indicating that deletion of the *dns* gene had no effect on bacterial survival under normal growth conditions. We observed that *dns* deletion led to a significant increase in the number of recovered transformants by approximately 100-fold compared with that of the wild type strain (Fig. 1C), thereby dramatically increasing the transformation efficiency (Fig. 1D). Furthermore, we established a highly efficient electroporation transformation method in the *dns* mutant by optimizing the electric field intensity (initial pulse voltages from 600 V to 1500 V). At an optimal pulse voltage of 900 V, the maximum transformation efficiency (1.3 × 10^4^ CFU/μg) was obtained for the *dns* mutant, which was 140-fold higher than that of the wild type strain (Fig. 1E). We further quantified the editing efficiency of the *dns* deletion by analyzing 47 colonies using colony PCR (Fig. S1). We sequenced the target region for the colonies and detected the desired sequence in all positive clones. The results indicated that the *dns* gene was deleted, with 44 of 47 colonies being correct (Fig. 1F), indicating a remarkably high editing efficiency (93.6%).

We sought to apply the dual-plasmid CRISPR-Cas9 editing system in the *dns*-deleted strain to generate deletions of other target genes with efficiencies of up to 89 ∼ 100%. As shown in Fig. 1F, we successfully deleted all target genes with high editing efficiencies (100∼89%). In addition, we further tested whether NT-CRISPR is applicable for integration into the genome. We found that the editing efficiency was slightly lower, with only 38 of 47 colonies carrying the desired Ptrc-*ScSAM2* integration (Fig. 1F). These data support the potential of the dual-plasmid CRISPR-Cas9 editing system for gene deletion and integration in *V. natriegens*.

### 3.2 Investigation of carbon source utilization and endogenous ergothioneine production by *V. natriegens* and the *dns*-*hutH* double-deletion strain V.nEgt01

**Fig. 2:**
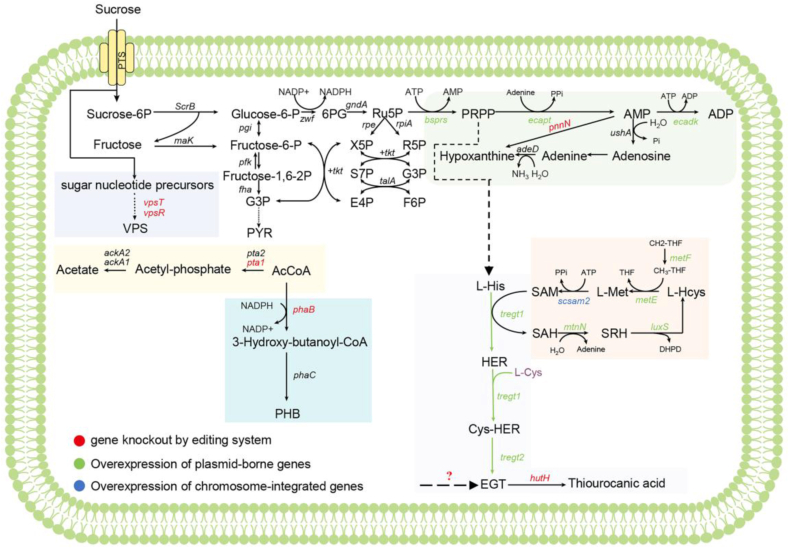
**Schematic of the metabolic pathway for producing metabolite precursors and byproducts and metabolic engineering strategies for efficient ergothioneine production from sucrose in *V. natriegens*.** The question mark indicates the existence of a currently undetermined endogenous ergothioneine synthesis pathway in *V. natriegens*. The green arrows indicate the heterologous ergothioneine synthesis pathway constructed in this study. PYR, pyruvate; AcCoA, acetyl-coenzyme A; 6PG, 6-phosphogluconate; X5P, xylose-5-phosphate; S7P, sedoheptulose-7-phosphate; E4P, erythrose-4-phosphate; R5P, ribose-5-phosphate; Ru5P, ribulose-5-phosphate; Fructose-1,6-2P, fructose-1,6-diphosphate; F6P, fructose-6-phosphate; Glucose-6-P, glucose-6-phosphate; G3P, glyceraldehyde-3-phosphate; PRPP, 5-phosphoribosyl-1-pyrophosphate; AMP, adenosine monophosphate; ADP, adenosine diphosphate; PHB, poly-β-hydroxybutyrate; L-His, l-histidine; L-Cys, l-cysteine; L-Met, l-methionine; SAM, S-adenosylmethionine; SRH, S-ribosylhomocysteine; SAH, S-adenosyl-l-homocysteine; L-Hcy, l-homocysteine; HER, l-histidine betaine; γ-GC-HER, γ-l-glutamyl-S-(hercyn-2-yl)-l-cysteine sulfoxide; Cys-HER, S-(hercyn-2-yl)-l-cysteine sulfoxide; EGT: ergothioneine; VPS, extracellular polysaccharide; PHB, polyhydroxybutyrate. See the text for more details.

An analysis of the genome revealed that *V. natriegens* ATCC14048 possesses a complete and efficient native sucrose utilization system [49]. We constructed a core sucrose metabolic model based on the annotated genes of *V. natriegens* (Fig. 2). In this model, sucrose is first transported into the cell via the phosphotransferase system (PTS) and is subsequently phosphorylated to sucrose-6-phosphate by the enzyme encoded by the sucrose-specific PTS transporter component enzyme IIA ScrA. Sucrose-6-phosphate is hydrolyzed by the sucrose-6-phosphate hydrolase ScrB into fructose and glucose-6-phosphate. Fructose is then phosphorylated by the fructokinase ScrK to yield fructose-6-phosphate, allowing both fructose-6-phosphate and glucose-6-phosphate to enter the central metabolic pathway. In addition, sucrose metabolism is typically accompanied by the production of byproducts such as extracellular polysaccharide, polyhydroxybutyrate and acetate. These byproduct pathways serve as target pathways for further metabolic engineering modifications to increase the production of ergothioneine (Fig. 2).

**Fig. 3:**
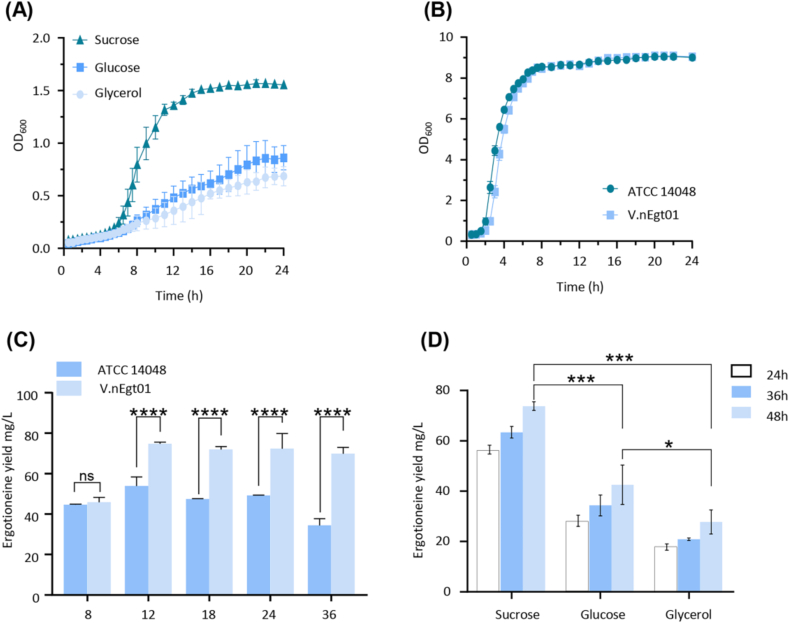
**Carbon source utilization and endogenous ergothioneine production in *V. natriegens* ATCC14048 and V.nEgt01.** (A) Growth rate assays of ATCC14048 grown on sucrose, glucose, or glycerol as the sole carbon source. (B) Growth rate assays of ATCC14048 and V.nEgt01. (C) Endogenous ergothioneine production by ATCC14048 and V.nEgt0 in shake flasks. (D) Effect of different carbon sources on endogenous ergothioneine production by V.nEgt01.

*V. natriegens* is capable of growing on various carbon sources, such as sucrose, glycerol, glucose, and arabinose [18]. The growth rate and ergothioneine productivity of this strain were compared in M9 minimal medium containing sucrose, glycerol, or glucose to identify the optimal sole carbon source. After 12 h, the growth on sucrose exceeded that on glucose by 2.4-fold and that on glycerol by 3-fold (Fig. 3A). In addition, our preliminary work indicates that low concentrations of ergothioneine are detected in the fermentation broth. Bioinformatics analysis revealed an intracellular ergothioneine degradation pathway in which histidine ammonia-lyase (HutH) cleaves ergothioneine by exploiting its structural similarity and breaking the C–N bond on its imidazole-like ring. In the *dns*-deficient genetic background, we constructed a *dns*-*hutH* double-deletion strain, V.nEgt01, using the dual-plasmid CRISPR-Cas9 editing system. As shown in Fig. 3B, V.nEgt01 exhibited normal cell growth similar to that of *V. natriegens* ATCC14048. After 48 h of cultivation in a shake flask, the concentration of ergothioneine in *V. natriegens* ATCC14048 gradually decreased to 30 mg/L from 52 mg/L. In contrast, the concentration of ergothioneine in V.nEgt01 remained almost unchanged and was 2.7-fold higher than that in *V. natriegens* ATCC14048 (Fig. 3C), suggesting that the *hutH* deletion substantially suppressed the degradation of ergothioneine. We also found that the ergothioneine titer of V.nEgt01 was 71 mg/L when sucrose was used as the sole carbon source, which was 1.73- and 2.7-fold higher than those obtained when glucose and glycerol were used, respectively (Fig. 3D). Although the endogenous ergothioneine concentration was too low to meet the demands of industrial-scale production, V.nEgt01 can serve as a chassis strain for further metabolic engineering modifications to maximize ergothioneine production.

### 3.3 Construction of heterologous ergothioneine biosynthesis pathways in the engineered chassis strain V.nEgt01

**Fig. 4:**
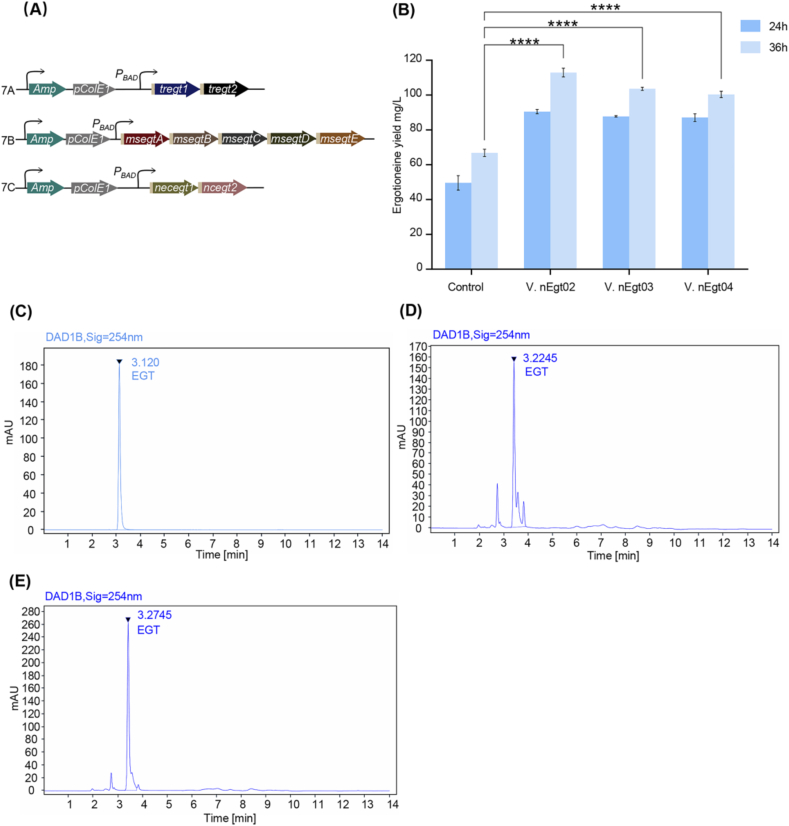
**Construction of heterologous ergothioneine biosynthesis pathways in the chassis strain V.nEgt01.** (A) Visualization of three recombinant plasmids (7A, 7B, and 7C) expressing the fungal pathway from *T. reesei* and *N. crassa* and the bacterial pathway from *M. smegmatis*, which were used for the construction of engineered strains. The expression of heterologous ergothioneine biosynthesis genes is driven by the arabinose-inducible promoter pBAD. (B) Heterologous ergothioneine biosynthesis in the engineered strain V.nEgt02 expressing TrEgt1 and TrEgt2 from *T. reesei*, V.nEgt 03 expressing NcEgt1–NcEgt2 from *N. crassa*, and V.nEgt 04 expressing MsEgtABCDE from *M. smegmatis*. The chassis strain V.nEgt01, which is able to produce endogenous ergothioneine, served as the control. (C) HPLC analysis of the ergothioneine standards (50 mg/L). (D) HPLC analysis of the endogenous ergothioneine produced by the engineered strain V.nEgt01. (E) HPLC analysis of the heterologous ergothioneine produced by the engineered strain V.nEgt02.

The biosynthetic pathways of ergothioneine are classified mainly into the fungal *egt1* and *egt2* pathways and the bacterial *egtABCDE* pathway (Fig. 2), but most reported studies with high ergothioneine yields have focused on the fungal pathway [10,11,50]. In this study, three recombinant plasmids (7A, 7B, and 7C) expressing components of the fungal pathway from *T. reesei* and *N. crassa* and components of the bacterial pathway from *M. smegmatis* were constructed (Fig. 4A) and introduced into the chassis strain V.nEgt01, which resulted in the engineered strains V.nEgt02, V.nEgt 03, and V.nEgt 04. As shown in Fig. 4B, compared with V.nEgt02 and V.nEgt03 which contain the fungal pathway, V.nEgt04 containing the bacterial pathway from *M. smegmatis* had a much lower yield. Markedly, V.nEgt02 containing TrEgt1 and TrEgt2 from *T. reesei* achieved an ergothioneine titer of 115 mg/L, which is 1.74-fold higher than that of V.nEgt01 with the endogenous pathway. HPLC analysis showed that the extracted samples displayed a predominant peak (254 nm) at a retention time of about 3 min, which was the same as that of the ERG standard (Fig. 4C, D, 4E). An analysis of the protein expression profile indicated that the expression of TrEgt1–TrEgt2 was very low in V.nEgt02 (Fig. S2), suggesting that the efficacy of the heterologous production of ergothioneine is limited mainly by low expression of the key enzymes TrEgt1 and TrEgt2 involved in the biosynthetic pathways.

### 3.4 Increased expression of TrEgt1 and TrEgt2 by the combination of promoters and RBSs with different strengths

**Fig. 5:**
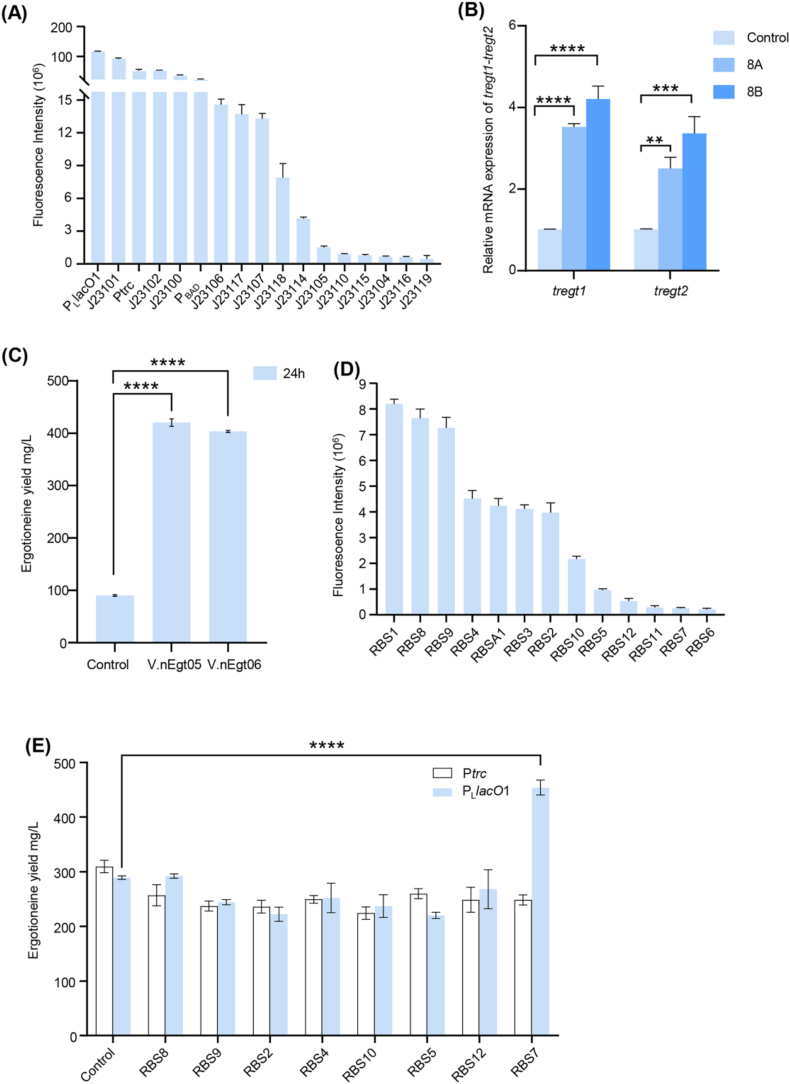
**Detection of Tregt1 and Tregt2 expression and ergothioneine yield in the engineered strain V.nEgt02 containing the combination of promoters and RBSs of different strengths.** (A) Effects of different promoter strengths on sfGFP fluorescence intensity in the *V. natriegens* wild type strain ATCC14048. (B) Quantitative RT‒PCR analysis of the relative expression levels of the *tregt1* and *tregt2* genes driven by the combination of the IPTG-inducible strong promoter Ptrc and the original RBS (plasmid 8A) and the combination of the superstrong P_L_*lacO1* promoter and the original RBS (plasmid 8B). Control, Plasmid 7A expressing TrEgt1 and TrEgt2 driven by the combination of the arabinose-inducible promoter pBAD and the original RBS. (C) Heterologous ergothioneine biosynthesis in the engineered strain V.nEgt05 containing plasmid 8A and V.nEgt06 containing plasmid 8B. V.nEgt02 containing plasmid 7A served as the control. (D) Effect of RBSs of different strengths on sfGFP fluorescence intensity in the *V. natriegens* wild type strain ATCC14048. (E) Ergothioneine yield resulting from combinations of promoters and RBSs of different strengths in the engineered strain V.nEgt05 containing plasmid 8A and V.nEgt06 containing plasmid 8B. Control, V.nEgt02 containing plasmid 7A with a combination of pBAD and the original RBS.

We constructed a customized and modular promoter–RBS component library to increase heterologous TrEgt1 and TrEgt2 expression. Our results demonstrated the utility of these promoters and RBSs in *V. natriegens*. As shown in Fig. 5A, the tested promoters mediated a wide dynamic range of expression, spanning approximately four orders of magnitude in sfGFP fluorescence intensity. Based on their relative strengths, these promoters were categorized into four tiers: weak (J23105, J23110, J23115, J23104, J23116, and J23119), medium (J23106, J23117, J23107, J23118, and J23114), strong (J23101, Ptrc, J23102, and J23100), and superstrong (P_L_*lacO1*).

By replacing the arabinose-inducible promoter pBAD in plasmid 7A, we combined the strong IPTG-inducible promoter P*trc* with the original RBS (plasmid 8A) and combined the superstrong P_L_*lacO1* promoter and the original RBS (plasmid 8B) to drive the expression of *tregt1* and *tregt2* (Fig. S3A). Plasmids 8A and 8B were introduced into V.nEgt02, resulting in the engineered strains V.nEgt05 and V.nEgt06, respectively. When the P*trc* and P_L_*lacO1* promoters were used to express *tregt1* and *tregt2* genes from the plasmids, a high level of the TrEgt1 and TrEgt2 expression in V.nEgt05 and V.nEgt06 compared with V.nEgt02 (Fig. S3B). These results are consistent with the results of the quantitative real-time RT‒PCR analysis of *tregt1* and *tregt2* expression, as shown in Fig. 5B. The two engineered strains were further tested in shake flasks. As shown in Fig. 5C, V.nEgt05 and V.nEgt06 produced 443 mg/L and 418 mg/L extracellular ergothioneine, respectively, which is approximately 4-fold higher than that of V.nEgt02, suggesting that the substitution of both P*trc* and P_L_*lacO1* substantially increased ergothioneine production.

The fluorescence output mediated by the 12 tested RBS elements exhibited a dynamic range spanning approximately two orders of magnitude (Fig. 5D). These RBSs were classified into three distinct strength tiers: weak (RBS5, RBS12, RBS11, RBS7, and RBS6), medium (RBS4, RBS1, RBS3, RBS2, and RBS10), and strong (RBS8 and RBS9). Furthermore, we chose plasmid 8A containing the IPTG-inducible strong promoter P*trc* and plasmid 8B containing the superstrong P_L_*lacO1* promoter to investigate the effect of RBS substitutions on ergothioneine production. The original RBS of the two plasmids was substituted with weak (RBS5, RBS12, and RBS7), medium (RBS4, RBS1, RBS2, and RBS10), and strong (RBS8 and RBS9) RBSs, respectively. The resulting plasmids were then transformed into V.nEgt02. A high level of ergothioneine production was observed when the original RBS was substituted with RBS7. The engineered strain V.nEgt08 containing the combination of the superstrong P_L_*lacO1* promoter and weak RBS7 achieved an ergothioneine titer of 469 mg/L, which was about 4-fold higher compared to that of V.nEgt02.

### 3.5 Effects of the consecutive knockout of byproduct pathways on ergothioneine production

The metabolism of ergothioneine in *V. natriegens* is typically accompanied by the production of byproducts, such as polyhydroxybutyrate (PHB), *Vibrio* polysaccharide (VPS) and acetate [42]. These byproduct pathways thus serve as target pathways for further metabolic engineering modifications. We tried to consecutively knock out the genes involved in the byproduct pathways in the engineered strain V.nEgt08 to further increase ergothioneine biosynthesis.

**Fig. 6:**
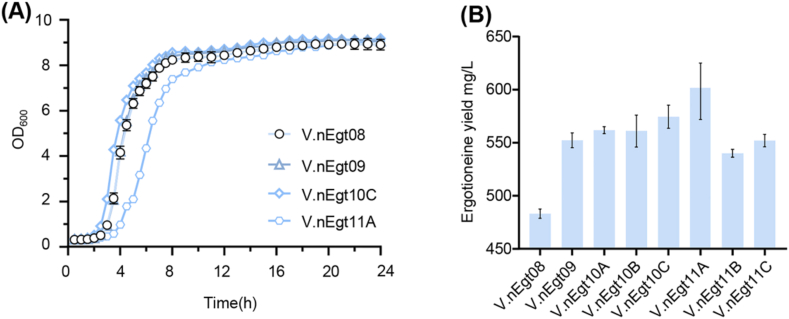
**Effects of the consecutive knockout of byproduct pathways on bacterial growth and ergothioneine production.** (A) Growth curves of different byproduct-deficient strains in shake flasks. circle, V.nEgt08 serving as a control; triangle, V.nEgt09 (△*phaB*); square, V.nEgt10C (△*vpsT*△*vpsR*); hexagon, V.nEgt11A (△*pta1*). (B) Comparison of ergothioneine production by different byproduct-deficient strains in shake flasks.

The determination of the genome nucleotide sequence led to the identification of a polyhydroxybutyrate (PHB) biosynthesis gene cluster in *V. natriegens*. The gene cluster includes three key genes, *phaA*, *phaB*, and *phaC*, which encode acetyl-CoA acetyltransferase, acetoacetyl-CoA reductase, and poly(3-hydroxyalkanoate) synthetase, respectively [22]. We knocked out the *phaB* gene encoding acetoacetyl-CoA reductase in *V. natriegens* to block PHB synthesis and simultaneously increase ergothioneine accumulation. After the *phaB* mutant V.nEgt09 was cultivated in shake flasks, no significant change in growth was observed compared with that of the parental strain (Fig. 6A). Sudan black staining and electron microscopy showed the presence of small lipid granules in the wild type strain but not in the *phaB* mutant V.nEgt09 (Fig. S4), indicating that the mutant lost the ability to synthesize PHB. As shown in Fig. 6B, the PHB-deficient strain V.nEgt09 can produce ergothioneine with a titer of 507 mg/L, representing a 9% increase in ergothioneine yield compared to V.nEgt08.

In *V. natriegens*, the gene cluster (*vpsI* and *vpsII*) is required for VPS synthesis, which is regulated by the upstream genes *vpsR* and *vpsT* [51,52]. The *vpsR* and *vpsT* genes were knocked out by consecutively deleting one gene after the other, resulting in three mutant strains, V.nEgt10A (△*vps*T), V.nEgt10B (△*vps*R), and V.nEgt10C (△*vps*T△*vps*R). No significant change in the growth of these strains was observed compared with that of the parental strain (Fig. 6A). In the LB3 solid plate assay, the wild type strain formed an opaque, wrinkled colony, and this colony was much larger than those of the three mutant strains, suggesting that it displayed significantly decreased VPS biosynthesis (Fig. S5). We found that among the three VPS-deficient strains, V.nEgt10C exhibited the most notable decrease in VPS biosynthesis (Fig. S5) and a 7% increase in ergothioneine yield compared to V.nEgt08 (Fig. 6B).

Acetate is synthesized by the Pta–AckA pathway in *V. natriegens*, in which two copies of the *pta* gene located on two separate chromosomes encode phosphotransacetylase [42,45,53]. Thus, we performed single and double knockouts in *pta1* and *pta2*, respectively, and measured the growth curves and acetate utilization of the resulting strains. The results show that when grown LB medium, the single and double knockout strains displayed similar growth rates to that of control (Fig. S6A). When grown at low and high concentrations of acetate (13 mM and 122 mM) as the sole carbon source, V.nEgt11A (△*pta2*) grow well, whereas growths of both V.nEgt11A (△*pta1*) and V.nEgt11C (△*pta1*△*pta2*) is significantly reduced during the logarithmic growth phase (Fig. S6B and S6C). These data indicated *pta1*, but not *pta2*, is involved in acetate utilization of *V. natriegens*, consistent with the previous report [45]. After fermentation, pH monitoring of the fermentation broth revealed that the pH of the *pta*-deleted strains was approximately 6.45, while the pH of the parental strain was 5.46 (Fig. S6D), suggesting that knocking out the acetic acid biosynthesis pathway can reduce the production of acetic acid, thereby increasing the pH of the fermentation broth. In addition, compared with the parental strain V.nEgt10C, the *pta1*-*pta2* double deletion led to decreased ergothioneine production (Fig. 6B). In contrast, the *pta1* mutant strain V.nEgt11A exhibited an ergothioneine concentration of 602 mg/L (Fig. 6B), which represents a 1.6-fold increase compared to that of V.nEgt08.

### 3.6 Engineering strategies to increase the supplies of SAM and ATP for ergothioneine biosynthesis

**Fig. 7:**
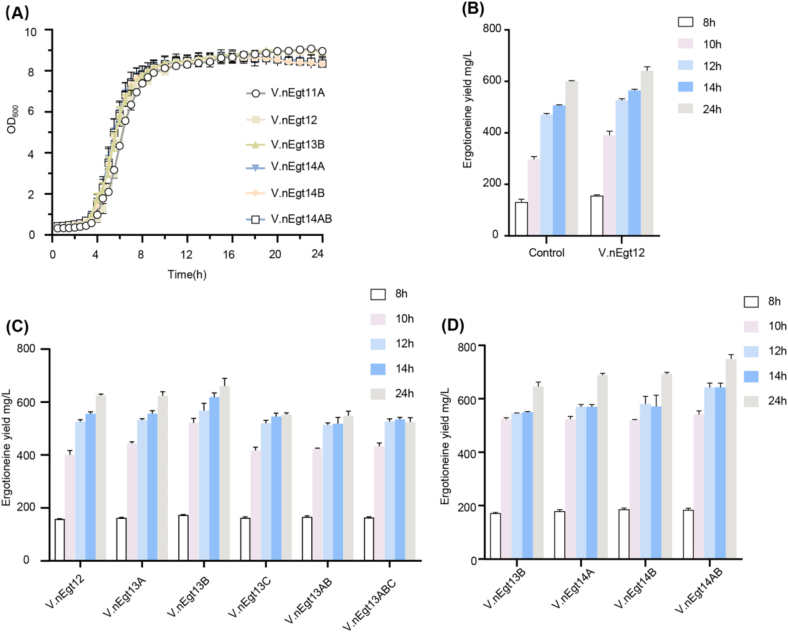
**Increasing the S-adenosylmethionine and ATP supplies for ergothioneine biosynthesis.** (A) Growth curves of different engineered strains and V.nEgt11A as a control. (B) Ergothioneine production by the engineered strain V.nEgt12 overexpressing ScSAM2 and V.nEgt11A as a control in shake flasks. (C) Ergothioneine production by V.nEgt13A (△*ushA*), V.nEgt13B (△*pnnN*), V.nEgt13C (△*adeD*), V.nEgt13AB (△*ushA* △*pnnN*), V.nEgt13ABC (△*ushA* △*pnnN* △a*deD* and V.nEgt12 as a control in shake flasks. (D) Ergothioneine production by V.nEgt14A coexpressing the *apt-adk* genes, V.nEgt14B coexpressing the *mtn-luxS-metE-metF* genes, V.nEgt14AB coexpressing the *apt-adk-mtn-luxS-metE-metF* genes, and V.nEgt13B as a control in shake flasks.

In the ergothioneine biosynthetic pathway, S-adenosylmethionine (SAM) serves as an indispensable methyl donor and is synthesized from methionine and ATP by methionine adenosyltransferase [54]. The SAM biosynthetic pathway and regulatory nodes can be engineered to increase the availability of intracellular SAM and thus increase the yield of target products. The biosynthesis of SAM from methionine and ATP is catalyzed by S-adenosylmethionine synthetase, which is encoded by both the SAM1 gene and the SAM2 gene in *S. cerevisiae* [55]. Many studies have focused on the use of SAM2 to increase SAM production either by the overexpression or heterologous expression of this target enzyme [56]. Accordingly, we integrated one copy of sc*sam2* into the genome of V.nEgt11A, resulting in a V.nEgt12 mutant strain. Our results revealed that V.nEgt12 overexpressing SAM2 exhibited normal cell growth and achieved a significant improvement in yield with a titer of 635 mg/L (Fig. 7A and B).

We knocked out the genes involved in ATP degradation, namely, *ushA,* encoding 5′-nucleotidase; *pnnN,* encoding the nucleotide 5′-monophosphate nucleosidase; and *adeD,* encoding adenosine deaminase, to block the ATP degradation pathways and increase the ATP supply, resulting in three single mutant strains (V.nEgt13A, V.nEgt13B, and V.nEgt13C), a double-deletion strain (V.nEgt13AB), and a triple-deletion strain (V.nEgt13ABC). These mutant strains had no adverse effects on bacterial growth or ergothioneine yield (Fig. 7A), with exception of V.nEgt13B, whose ergothioneine yield increased by 14% (Fig. 7C). Plasmid 19A3 harboring ATP biosynthetic pathway genes (*apt-adk*), plasmid 21B harboring SAM cycle genes (*mtn-luxS-metE-metF*), and plasmid 23A harboring both ATP biosynthetic pathway genes and SAM cycle genes were constructed to further optimize V.nEgt13B. These plasmids were individually transformed into V.nEgt13B, resulting in three engineered strains, V.nEgt14A. V.nEgt14B, and V.nEgt14AB. In shake flasks, V.nEgt14AB produced a maximum ergothioneine titer of 711 mg/L (Fig. 7C), representing a 10-fold increase compared with that of the chassis strain V.nEgt01. Thus, the best-performing engineered strain, V.nEgt14AB, was used for subsequent fermentation experiments.

### 3.7 Non-sterile fed-batch fermentation for the efficient production of ergothioneine

**Fig. 8:**
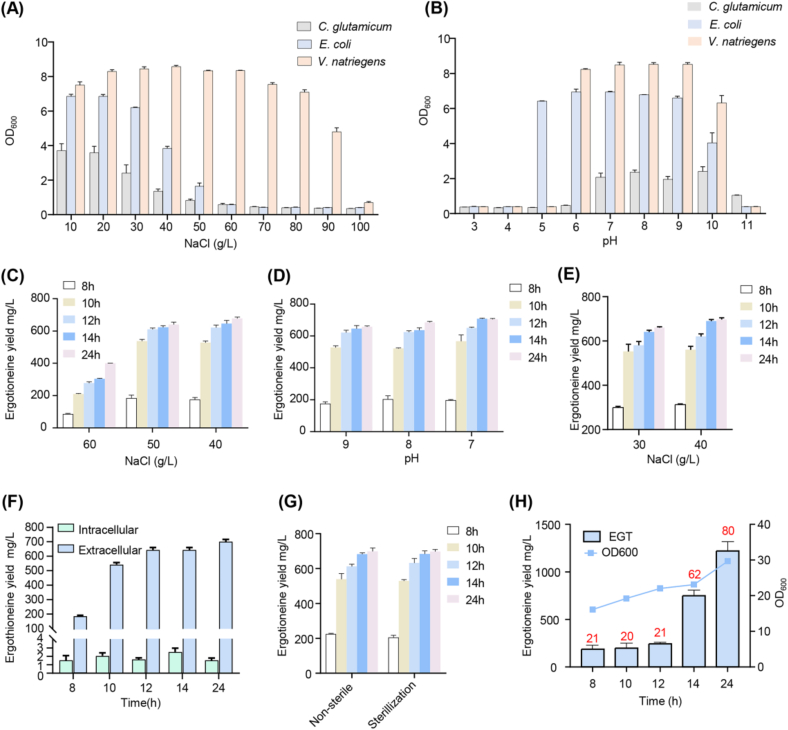
**Non-sterile fermentation with sucrose feeding for the efficient production of ergothioneine.** (A) Growth curve of the best-performing engineered strain, V.nEgt14AB, in M9 medium at different pH values in shake flasks. Two traditional model strains, *C. glutamicum* ATCC 13032 and *E. coli* MG1655, served as controls. (B) Growth curve of V.nEgt14AB in M9 medium containing various NaCl concentrations in shake flasks. *C. glutamicum* ATCC 13032 and *E. coli* MG1655 served as the controls. (C) Ergothioneine yield of V.nEgt14AB grown in M9 media supplemented with 40, 50 and 60 g/L NaCl at pH 7 in shake flasks. (D) Ergothioneine yield of V.nEgt14AB grown in M9 medium supplemented with 40 g/L NaCl when the pH was 7, 8, and 9. (E) Ergothioneine yield of V.nEgt14AB grown in M9 media supplemented with 30 and 40 g/L NaCl at pH 7 in shake flasks. (F) The content of both intracellular and extracellular ergothioneine in shake flasks. (G) Ergothioneine yield of V.nEgt14AB under non-sterile and sterile fermentation conditions in shake flasks. (H) Ergothioneine yield of V.nEgt14AB under non-sterile fed-batch fermentation conditions in a 2-L bioreactor. The numbers in red and above the column chart indicate the content of extracellular ergothioneine (mg/g DCW).

Salinity and pH are key factors for microbial growth and fermentation and can be controlled to favor the growth of desired strains under non-sterile conditions [57]. In this study, we employed M9 as a basal medium to evaluate the effects of NaCl and pH on the growth and ergothioneine production of the best-performing engineered strain, V.nEgt14AB. First, we measured the growth curve of V.nEgt14AB grown in M9 medium supplemented with various NaCl concentrations and with different pH values in shake flasks. The results showed that V.nEgt14AB exhibited normal cell growth when the NaCl concentration was between 40 g/L and 70 g/L or when the pH was between 6 and 9. As shown in Fig. 8A and B, V.nEgt14AB grew vigorously when the NaCl concentration was set to 80 g/L from 30 g/L or a pH of 10 to 5, but the growth of *C. glutamicum* ATCC 13032 and *E. coli* MG1655 was clearly inhibited in the presence of 30 g/L NaCl (equivalent to the salinity of seawater) and almost completely stopped in the presence of 60 g/L NaCl. Such superior salinity tolerance of V.nEgt14AB lays a foundation for nonsterilized fermentation in high-salinity media where the growth of other contaminating bacteria is largely suppressed, thereby reducing the cost and simplifying the industrial bioprocess.

Therefore, we employed a rational design approach to identify the optimal combination of NaCl concentrations and pH values for ergothioneine produced by V.nEgt14AB in shake flasks. Our results showed that at pH 7, V.nEgt14AB produced a higher ergothioneine titer in M9 medium containing 40 g/L NaCl than those obtained at 50 and 60 g/L NaCl (Fig. 8C). When the NaCl concentration was set to 40 g/L, V.nEgt14AB produced higher ergothioneine titers at pH 7 than those obtained at pH 8 and pH 9 (Fig. 8D). In addition, we compared the effect of 40 g/L NaCl on ergothioneine yields with that of 30 g/L NaCl, a salt concentration currently used in non-sterile fermentation. The results indicated that the ergothioneine yield at 40 g/L NaCl was higher than that obtained at 30 g/L NaCl (Fig. 8E).

To determine the correlation between cell growth and dry weight as well as the ergothioneine synthesis amount, fermentation broths were taken at regular intervals to determine the optical density (OD), and then the cell debris and the supernatant obtained after centrifugation and filtration were used to measure dry cell weight (DCW) and ergothioneine content. Our results showed that during non-sterile fed-batch fermentation, the engineered strain V.nEgt14AB grows rapidly, and DCW is positively correlated with the yield of ergothioneine. After 24 h of fermentation, the content of ergothioneine increased to 80 mg/g DCW from approximately 20 mg/g DCW in the initial stage of fermentation (Fig. S7). We also measured the content of both intracellular and extracellular ergothioneine in shake flasks. During the 24-h fermentation in shake flasks, the intracellular ergothioneine content was extremely low, only approximately 2 mg/L, whereas the ergothioneine content in the fermentation supernatant increased to 1200 mg/L from approximately 300 mg/L, indicating that the vast majority of the ergothioneine synthesized by the engineered strain V.nEgt14AB was secreted outside the cell (Fig. 8F). Furthermore, ergothioneine yield levels between the non-sterile and sterile fermentations were compared, and no difference in yield was observed between the two fermentation methods in shake flasks (Fig. 8G).

Fed-batch fermentations of engineered *V. natriegens* were performed in a 2 L bioreactor under high-salinity, non-sterile conditions. The seed culture was inoculated at 1% (v/v) into non-sterile M9 medium, in which the NaCl concentration was adjusted to 40 g/L. The cultivation conditions were as follows: temperature, 37 °C; dissolved oxygen, 100%; and agitation speed, 300 rpm. The pH was automatically controlled at 7.0. When a noticeable increase in the dissolved oxygen content was observed, a 500 g/L sucrose feed solution was added. Fermentation broths were taken at regular intervals to determine OD, and then the cell debris and the supernatant obtained after centrifugation and filtration were used to measure DCW and ergothioneine content. Our results showed that during non-sterile fed-batch fermentation, the engineered strain V.nEgt14AB grows rapidly and DCW is positively correlated with the yield of ergothioneine while the content of ergothioneine increased to 80 mg/g DCW from approximately 20 mg/g DCW in the initial stage of fermentation (Fig. 8H). After 24 h of fermentation, V.nEgt14AB achieved an ergothioneine titer of 1.2 g/L with a productivity of 50 mg/L/h, representing a 1.7-fold increase compared with that in shake flasks. These data suggested that non-sterile fermentation with sucrose feeding by *V. natriegens* may help reduce the costs of both fermentation media and energy.

## 4 Discussion

Ergothioneine (EGT) is a unique natural antioxidant that is well known for its strong anti-inflammatory, anti-apoptotic, anti-aging, and metal-chelating properties. Natural edible mushrooms possess EGT synthesis pathways and are commonly used in ergothioneine production. Various strategies have been employed to enhance cell growth and ergothioneine yield in macrofungal fermentation, including optimizing carbon and nitrogen sources, adjusting temperature, and timing the harvest appropriately. Tepwong et al. [58] utilized *Lentinula edodes* mycelium for immersion fermentation in a synthetic medium, achieving a maximum ergothioneine production of 0.913 mg/L on the 15th day. With the advancement in synthetic biology, ergothioneine biosynthesis pathways have been genetically engineered into traditional model strains*,* including *E. coli*, *C. glutamicum*, and *S*. *cerevisiae*. Overexpression of truncated Egt1 from *N. crassa*, along with the *egtD* and *egtE* genes from *M. smegmatis* in *E. coli* resulted in a final ergothioneine yield of 5.4 g/L, after 96 h of fermentation [59]. By optimizing sulfur assimilation and pentose phosphate pathways and increasing the accumulation of l-histidine and l-cysteine precursors in *C. glutamicum*, a yield of 264 mg/L ergothioneine was achieved after 36 h of fermentation [60]. Besides genetic modifications, the application of ultraviolet and lithium chloride for random mutagenesis in *S. pombe* led to the generation of a highly efficient ergothioneine synthesis mutant, OMK-79, achieving a yield of 12.5 g/L after 148 h of optimized culture, which represents the highest level of ergothioneine production in yeast chassis strains [61]. Nevertheless, the excessively long production cycle and high production costs remain major obstacles for the industrial-scale bioproduction of ergothioneine or other valuable chemicals. In this study, we developed a dual-plasmid CRISPR-Cas9 editing system and explored the feasibility of non-sterile fed-batch fermentation from low-cost sucrose for the efficient biosynthesis of ergothioneine, which also provides a pivotal starting point for the use of *V. natriegens* as an efficient and economic platform to produce various value-added chemicals.

**Fig. 9:**
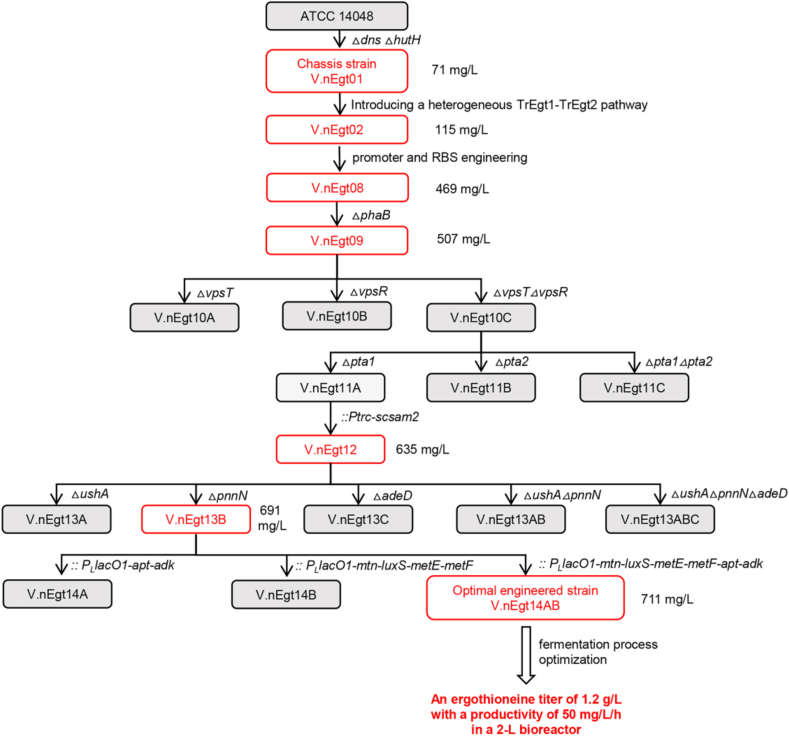
**Schematic diagram of efficient ergothioneine biosynthesis by engineered *V. natriegens* through multiple engineering strategies and optimization of the fermentation process.** The applicability of the dual-plasmid CRISPR-Cas9 editing system for gene deletion and integration in *V. natriegens* chromosomal regions was confirmed. Using this method, a set of engineered strains was successfully generated for efficient ergothioneine biosynthesis. Strains in the red boxes represent engineered strains displaying significantly increased production of ergothioneine. The ergothioneine yield (mg/L) listed next to the red boxes represents the fermentation results from engineered strains grown in shake flasks. See the text for more details.

*V*. *natriegens* is recognized as a next-generation industrial strain for the bioproduction of various value-added chemicals because of its fast growth, broad substrate scope, and superior environmental adaptability. Its extraordinarily short doubling time of less than 10 min provides a theoretical foundation for achieving high volumetric productivity [62]. However, such rapid growth can exacerbate competition for core cellular resources (e.g., ATP and ribosomes) between the heterologous pathway and native host metabolism [62,63]. In addition, the cloning process in *V. natriegens* is less robust than that in highly engineered *E. coli*, mainly because of the drastically lower transformation efficiency of *V. natriegens* [16]. To improve the efficiency of genetic manipulation, we constructed a *dns* mutant strain and its electrotransformation efficiency was approximately 100-fold higher than that of the wild type strain. We further established a dual-plasmid CRISPR-Cas9 editing system and confirmed its applicability for the deletion and integration of *V. natriegens* chromosomal regions. Using the *dns*-deficient genetic background, we constructed a *dns*-*hutH* double-deletion strain, V.nEgt01, which could serve as a chassis strain for further metabolic engineering modifications. Initially, we introduced the heterologous ergothioneine synthesis genes *tregt1* and *tregt2* and successfully generated the engineered strain V.nEgt02, which is capable of producing ergothioneine (115 mg/L). Secondly, the combination of the superstrong P_L_*lacO1* promoter and weak RBS7 in V.nEgt08 subsequently led to the optimal expression level of TrEgt1 and TrEgt2, thereby markedly increasing ergothioneine production, with a titer of 466 mg/L, which represents a 60% increase compared with that in V.nEgt02. Thirdly, we deleted consecutively genes encoding components of byproduct pathways in V.nEgt11A to increase ergothioneine biosynthesis. Fourthly, engineering strategies were employed to increase the supplies of SAM and ATP for ergothioneine biosynthesis, and the resulted strain V.nEgt14AB produced a maximum ergothioneine titer of 711 mg/L. Lastly, the best-performing engineered strain, V.nEgt14AB, was selected for non-sterile fed-batch fermentation and achieved an ergothioneine titer of 1.2 g/L. A schematic diagram of the efficient biosynthesis of ergothioneine by *V. natriegens* through multiple engineering strategies and optimization of the fermentation process is illustrated in Fig. 9.

As mentioned above, *V. natriegens* is characterized by fast growth, a broad substrate scope, and superior environmental adaptability, most likely resulting in high fermentation productivity. The application of *V. natriegens* will be significantly expanded to allow direct nonsterilized fermentation, which favors low-cost, scaled-up industrial production. Our results demonstrated the feasibility of *V. natriegens* for nonsterilized ergothioneine production in high-salt media with low-cost sucrose as the sole carbon source, indicating that *V. natriegens* has an extra economic advantage over other bacteria for ergothioneine production. The biosynthesis of ergothioneine has been predominantly explored in traditional model strains, such as *E. coli* and *C. glutamicum*, typically using glucose or glycerol as carbon sources [10,64]. However, these industrial workhorses present significant metabolic constraints for the use of alternative feedstocks. Specifically, *E. coli* K-12 strains lack a complete sucrose metabolic pathway [65], and *C. glutamicum* generally lacks efficient native mechanisms for sucrose utilization [66], which collectively limits access to cheaper carbon substrates. From an economic standpoint, sucrose is a highly attractive alternative, as it is an abundant, price-stable agricultural commodity whose market price is considerably lower than that of conventional fermentation carbon sources, such as glucose and glycerol [67]. Intriguingly, the genome of *V. natriegens* harbors a complete sucrose metabolic gene cluster (*scr* regulon), suggesting an inherent capacity for efficient sucrose assimilation [25]. Apart from its adaptability to less expensive organic substrates, the superior salt tolerance of *V. natriegens* presents another advantage for ergothioneine bioproduction under high-salinity, non-sterile fermentation conditions, offering unique advantages for cost-effective bioprocessing. We found that the growth of *E. coli* was obviously decreased in the presence of 30 g/L NaCl, whereas *V. natriegens* vigorously grew in the presence of NaCl concentrations ranging from 40 g/L to 70 g/L. This superior salt tolerance of *V. natriegens* provides a foundation for non-sterile fermentation in high-salinity media where the growth of microbial contaminants is substantially suppressed.

In this study, we found that the *pta1* single knockout increased ergothioneine yield, but both the *pta2* single knockout and double knockout resulted in a decrease. One important reason is the complexity of acetate metabolism in *V. natriegens.* Numerous investigations have demonstrated that acetate typically occurs as a byproduct of overflow metabolism through phosphotransacetylase (Pta) and acetate kinase (AckA) pathway in many bacteria. In addition, *V. natriegens* has been demonstrated to grow well on acetate as a sole carbon source, but its associated growth kinetics and metabolic pathways have not been described (Establishing *Vibrio natriegens* as a high-performance host for acetate-based poly-3-hydroxybutyrate production [45]). Most strikingly, we found that the *V. natriegens* genome is predicted to possess two copies of each of *pta* (RS03410 and RS22305), *ackA* (RS03415 and RS21805), and *acsA* (RS14410 and RS21410) genes, which encode phosphotransacetylase, acetate kinase, and Acetyl-CoA synthetase, respectively. Such a genetic feature involving multiple copies of acetate metabolic genes might not only contribute to *V. natriegens*’ natural efficiency for rapid acetate utilization but also reduce the risk of loss of a critical component of acetate metabolic pathway. On the other hand, this functional redundancy significantly increases the difficulty of studying gene function through the phenotypic analysis of mutant strains. Nonetheless, we speculate that there might be another possibility that could lead to a decrease in ergothioneine yield of both the *pta2* single knockout strain and double knockout strain. The reasonable and worthy possibility for further research regarding such a phenomenon is that *pta2* may also have unknown distinctive roles in other cellular metabolisms that might affect ergothioneine biosynthesis.

S-Adenosylmethionine (SAM) serves as a direct precursor of the ergothioneine biosynthetic pathway and is synthesized from methionine and ATP by methionine adenosyltransferase [54]. The primary metabolic engineering strategies for increasing the supplies of S-adenosylmethionine (SAM) and ATP in microbial cell factories involve the multidimensional optimization of their synthesis, regeneration, and recycling pathways. Common approaches to enhance the SAM cycle include the exogenous supplementation of methionine precursors [68]; overexpression of SAM synthetases, such as MetK from *E*. *coli* and its high-activity variant MetK I303V [69], or SAM2 from *S*. *cerevisiae* [70]; and the systematic reconstruction of the SAM regeneration cycle [68]. The core of the regeneration cycle lies in the efficient degradation of the inhibitory metabolite S-adenosylhomocysteine (SAH), where the cleavage pathway catalyzed by the enzymes Mtn and LuxS has proven to be superior to the one-step hydrolysis pathway [68]. Concurrently, enhancing the 5-methyltetrahydrofolate (5-MTHF) cycle by overexpressing enzymes such as MetF provides an adequate supply of methyl donors for SAM regeneration [68]. Strategies designed to increase the ATP supply focus on constructing efficient regeneration systems, such as introducing modules comprising acetate kinase Ack and adenylate kinase Adk in *E. coli*, which regenerate ATP using the low-cost substrates acetyl phosphate (ACP) and AMP [70]. Moreover, the simultaneous knockout of genes such as *amn*, *adeD*, and *ushA* blocks the degradation and loss of ATP/adenine [68], in combination with the overexpression of *apt* and *adk* to strengthen the adenine–ATP cycle, which recycles adenine from SAH cleavage products back into ATP [68]. Furthermore, optimizing central metabolism (e.g., introducing the heterologous phosphoketolase Xfpk–Pta pathway [69]) and increasing the supply of NADPH (e.g., expressing GapC and Pos5c [69]) can indirectly improve the cellular energy status. These integrated strategies have shown broad applicability and high efficiency in the production of various high-value compounds, such as ferulic acid [68], vanillin [69], and melatonin [68], providing a versatile and robust metabolic engineering solution for cofactor-dependent biosynthesis. The results obtained in this study, such as those from the gene deletion and integration experiments, indicated that the yield of ergothioneine observably increased when S-adenosylmethionine and ATP availability were engineered. In addition, we speculate that steps in the upstream methionine biosynthesis pathway and in the downstream SAM regeneration pathway appear to limit SAM utilization by heterologous methyltransferases, and these steps should receive greater initial focus in future studies that aim to understand and improve SAM biosynthesis and regeneration in *V. natriegens*. The strategies described in this work are likely to improve the production of other desired bioactive compounds by *V. natriegens*.

In metabolic engineering, a strong promoter paired with a strong RBS is the conventional logic for increasing protein expression, unless inclusion bodies form or metabolic burden is excessive. However, due to the complexity of biological systems and their metabolic regulation, especially in the heterologous synthesis of active substances such as ergothioneine, the excessive gene expression often results in a decrease in the yield of target products, rather than the higher the expression, the higher the yield. The reasons for this seemingly abnormal phenomenon are quite complex. A reasonable explanation is that excessive gene expression might cause an imbalance in various metabolisms within the cells, thereby leading to a decrease in the yield of target products. This would become an interesting topic for future studies. In this study, the engineered strain V.nEgt08 containing the combination of the superstrong P_L_*lacO1* promoter and weak RBS7 achieved an ergothioneine titer of 469 mg/L, which was much higher than that of all the other combinations of promoters and RBSs of different strengths, strongly indicating that the combined effect is unpredictable. As pointed out by Zhang B et al., among 9 genetic modules, the module of G^H^B^M^D^M^E^M^ was the most effective combination for production of shikimic acid synthesis in *C. glutamicum* [71]. This is also the view of Nowroozi et al., who stressed that a particular RBS can be strong when used with one gene but weak when used with another gene, making the prediction of protein production difficult [72]. More recently, Yue et al. demonstrated the successful application of systematic optimization of promoter strength and RBS elements to enhance biosynthesis of 6′-sialyllactose in *E*. *coli*, and also found that a descending RBS strength gradient in polycistronic systems substantially improves both protein expression and translational efficiency [73]. We anticipated that with the development of a series of high-throughput screening technology, it should be possible to test many more combinations of promoters and RBSs of different strengths for all the genes of interest in the pathway to achieve an optimal balance between the expression and metabolism.

Our results also revealed one significant physicochemical barrier that affected catalytic efficiency during the transition of engineered strains from a shake flask to a 2-L bioreactor. Under high-cell-density fermentation conditions, *V. natriegens* strains exhibited significant pronounced cellular autoaggregation. This protective behavior, often mediated by VPS and induced by environmental stresses such as high substrate concentrations [74], led to the formation of cellular aggregates and thus restricted mass transfer efficiency. Therefore, we knocked out two key VPS regulatory genes, *vpsR* and *vpsT*, but found that the knockout of these genes did not completely suppress aggregation, suggesting that the regulatory network underlying cellular autoaggregation in *V. natriegens* may be more complex than expected. Our results also showed that *V. natriegens* growth rapidly shifted from the logarithmic growth phase to the stationary phase within 24 h in shake flasks, while the logarithmic growth phase was sustained for up to 72 h in the 2-L bioreactor, accompanied by the efficient production of ergothioneine. In addition, substrate excess or depletion from batch feeding likely triggered a premature transition to a nonproductive state. Future pilot-scale studies must prioritize elucidating the physiological responses of *V. natriegens* at high cell densities, for instance, investigating whether quorum sensing triggers carbon flux redirection [26]. As reported for ergothioneine production in *C*. *glutamicum*, subtle physiological shifts can drastically alter metabolic output [75]. Consequently, the industrial application of *V. natriegens* will necessitate an integrated approach combining systems biology analysis with advanced process engineering. Implementing dynamic control strategies (e.g., pH or dissolved oxygen-dependent feeding) will be key to co-optimizing biomass formation and product biosynthesis, ultimately achieving efficient production [76,77].

## 5 Conclusion

In this study, a dual-plasmid CRISPR-Cas9 editing system with editing efficiencies of up to 100% was developed and further metabolically engineered to fully harness the potential of *V. natriegens* for ergothioneine production. Initially, 0.619 g/L ergothioneine was obtained from the chassis strain through the construction of a heterologous ergothioneine biosynthetic pathway. We subsequently combined the superstrong P_L_*lacO1* promoter and weak RBS7 to drive the expression of a heterogeneous TrEgt1–TrEgt2 pathway. In addition, several key genes involved in the SAM cycle and the ATP synthesis pathway were overexpressed, and genes encoding essential ATP-degrading enzymes were deleted, which led to the best-performing engineered strain V.nEgt14AB with a high ergothioneine yield (711 mg/L) in shake flasks. We further explored the feasibility of non-sterile fed-batch fermentation for the efficient biosynthesis of ergothioneine from low-cost sucrose. The engineered strain V.nEgt14AB achieved maximal production of ergothioneine (1.2 g/L) in a 2-L bioreactor. This study not only represents the first successful example of engineered *V. natriegens* to synthesize ergothioneine but also provides a pivotal starting point for the use of *V. natriegens* as an efficient and economic platform, with the goal of producing various value-added chemicals.

## CRediT authorship contribution statement

**Xinhui Liang:** Writing – review & editing, Writing – original draft, Validation, Methodology, Formal analysis, Data curation. **Yue Wang:** Validation, Methodology, Formal analysis, Data curation. **Yifei Lv:** Methodology, Formal analysis, Data curation. **Chaoyong Huang:** Methodology, Investigation, Formal analysis, Conceptualization. **Zhenbang Huang:** Project administration, Investigation, Data curation. **Jinfeng Wei:** Methodology, Data curation. **Shijie Jiang:** Investigation, Formal analysis, Data curation. **Zhiyang Dong:** Supervision, Investigation, Formal analysis, Conceptualization. **Zhengfu Zhou:** Methodology, Investigation, Formal analysis, Data curation. **Min Lin:** Writing – review & editing, Supervision, Funding acquisition, Conceptualization.

## Declaration of competing interest

The authors declare the following financial interests/personal relationships which may be considered as potential competing interests: Chaoyong Huang, Zhenbang Huang and Zhiyang Dong are currently employed by Shenzhen Siyomicro Bio-Tech Co., Ltd.
