# Supplementary material for: Metabolic engineering of Vibrio natriegens for the efficient biosynthesis of ergothioneine from sucrose using non-sterile fed-batch fermentation
Source: Synth Syst Biotechnol. 2026 Jun 12;14:498–514. doi: 10.1016/j.synbio.2026.04.041 (PMC13276445; doi:10.1016/j.synbio.2026.04.041)
Supplement: Multimedia component 1 [file mmc1.docx]

**Metabolic engineering of** ***Vibrio natriegens*** **for** **the efficient biosynthesis of ergothioneine from sucrose using nonsterile fed-batch fermentation**

Xinhui Liang^a^, Yue Wang^b^, Yifei Lv^b^, Chaoyong Huang^c^, Zhenbang Huang^c^, Jinfeng Wei^b^, Shijie Jiang^a^, Zhiyang Dong^c*^, Zhengfu Zhou^d*^, Min Lin^b*^

^a^ Engineering Research Center of Biomass Materials, Ministry of Education, College of Life Sciences and Agri-forestry, Southwest University of Science and Technology, Mianyang, Sichuan 621010, PR China

^b^ Food Laboratory of Zhongyuan, College of Agriculture, Henan University, Kaifeng 475001, P. R.China

^c^ **Shenzhen Siyomicro Bio-Tech Co., Ltd.** Shenzhen**,** Guangdong**, 518057,** P. R.China

^d^ Key Laboratory of Agricultural Microbiome (MARA), Biotechnology Research Institute, Chinese Academy of Agricultural Sciences, Beijing 100081, PR China

^*^ Corresponding author. E-mail address: zhouzhengfu@caas.cn (Z. Zhou), dongzy@im.ac.cn (ZY. Dong) and [linmin@henu.edu.cn](mailto:linmin@henu.edu.cn) (M. Lin)

| Strain/plasmid | Description | Sources |
| --- | --- | --- |
| ATCC 14048 | / | purchase |
| ATCC 13032 | / | Our laboratory |
| MG 1655 | / | Our laboratory |
| V.nEgt01 | ATCC 14048 deficient in *dns* and *hutH* | This study |
| V.nEgt02 | V.nEgt01 harboring the plasmid 7A  (Introduce heterogeneous approaches *TrEgt1-TrEgt2*) | This study |
| V.nEgt08 | promoter and RBS engineering on V.nEgt02 | This study |
| V.nEgt09 | V.nEgt08 deficient in *phaB* | This study |
| V.nEgt10A | V.nEgt09 deficient in *vpsT* | This study |
| V.nEgt10B | V.nEgt09 deficient in *vpsR* | This study |
| V.nEgt10C | V.nEgt09 deficient in *vpsT* and *vpsR* | This study |
| V.nEgt11A | V.nEgt10C deficient in *pta1* | This study |
| V.nEgt11B | V.nEgt10C deficient in *pta2* | This study |
| V.nEgt11C | V.nEgt10C deficient in *pta1* and *pta2* | This study |
| V.nEgt12 | V.nEgt11A *integrated with SAM2 from S. cerevisiae* | This study |
| V.nEgt13A | V.nEgt11 deficient in *ushA* | This study |
| V.nEgt13B | V.nEgt11 deficient in *pnn*N | This study |
| V.nEgt13C | V.nEgt11 deficient in *adeD* | This study |
| V.nEgt13AB | V.nEgt11 deficient in *pnnN* and *ushA* | This study |
| V.nEgt13ABC | V.nEgt11 deficient in *pnnN,* *ushA* and *adeD* | This study |
| pUC19 | pBR322-Amp | Our laboratory |
| pColE1 | vector for gene expression pColE1-pBAD-Amp | Our laboratory |
| p15A | vector for gene expression p15A-P_L_lacO1-Kan | Our laboratory |
| 7A | pColE1-pBAD-Amp-*TrEgt1*-*TrEgt2* | Our laboratory |
| 7B | pColE1-pBAD-Amp-*MsEgtABCD*E | Our laboratory |
| 7C | pColE1-pBAD-Amp-*NcEgt1*-*NcEgt2* | Our laboratory |
| 8A | pColE1-Ptrc-Amp-*TrEgt1*-*TrEgt2* | This study |
| 8B | pColE1-P_L_lacO1-Amp-*TrEgt1*-*TrEgt2* | This study |
| 8B_RBS7_ | pColE1-P_L_lacO1-Amp-RBS7-*TrEgt1*-*TrEgt2* | This study |
| 8B_5_ | pColE1-P_L_lacO1-RBS7-Amp-*TrEgt1*-*TrEgt2*-RBS1-*mtn* | This study |
| 19A3 | p15A-P_L_lacO1-Kan-*Bsprs*-*apt*-*adk*-Kan | This study |
| 21B | p15A-P_L_lacO1-Kan-*mtn*-*luxS*-*metE*-*metF*-Kan | This study |
| 23A | p15A-P_L_lacO1-Kan-*mtn*-*luxS*-*metE*-*metF*-*Bsprs*-*Ecadk*-*Ecapt*-Kan | This study |
| p15A-Cas9 | p15A-P_BAD_-*Cas9*-Ptrc-*λRed*-*SacB*-Kan | Our laboratory |
| p15A-sgRNA | p15A-sgRNA-*upp*-Amp | This study |
| pColE1-Cas9 | pColE1-P_BAD_-*Cas9*-Ptrc-*λRed*-*SacB*-Kan | This study |

**Table S1** Strains and plasmids used in this study.

**Tabel .S2** Primers used in this study

| **Primers** | **Sequence** |
| --- | --- |
| *dns*-U-F | caaaacctacccagccga |
| *dns*-U-R | gtcgcctcgtgaaatcac |
| *dns*-D-F | cggcactggatagtgcaag |
| *dns*-D-R | acccaagcgttcttgcaa |
| *araA*-U-F | gcatggaaaccttcctgc |
| *araA*-U-R | ctggggagaaagtgtgca |
| *araA*-D-F | gttcccaaagcgcgtgtg |
| *araA*-D-R | ggggaaccgacccacata |
| *hutH*-U-F | tccaaatcagacaaatgtgagct |
| *hutH*-U-R | agatgcgtttcttcaggtg |
| *hutH*-D-F | cacctaacgcgtgcgttc |
| *hutH*-D-R | gaagtcggcacgctgtca |
| *phaB*-U-F | cattttattcccatcgcggac |
| *phaB*-U-R | gcttcttagtgagtgatgcagg |
| *phaB*-D-F | ttgtttgtgagatctaaatcaataatacgaaa |
| *phaB*-D-R | cgttccaagcttctgcgg |
| *vpsT*-U-F | gaataaagacagttaatagttcaatgaatatatttaggt |
| *vpsT*-U-R | ttcaacagcgatgattgtagact |
| *vpsT*-D-F | aacatctttaagaagattgaagtgaaaaatagag |
| *vpsT*-D-R | accgagttggaatgcattca |
| *vpsR*-U-F | gaatcaagtcaaacgtgcagta |
| *vpsR*-U-R | cggttaaattgtaagctctggtg |
| *vpsR*-D-F | ggcagatcgtatcagcacc |
| *vpsR*-D-R | cctttaatttaaaccaattcagacaattgc |
| *pta1*-U-F | ggatatggagacggtgtgaa |
| *pta1*-U-R | cagaagtaattcttttctctgcttga |
| *pta1*-D-F | ctcacgagctacaacttcagt |
| *pta1*-D-R | gaaacaggcctacctatcttcaaag |
| *pta2*-U-F | gcaagaatggcgagtaacg |
| *pta2*-U-R | tggaagaacctaaagtggcc |
| *pta2*-D-F | atcctttccatcgccttcat |
| *pta2*-D-R | ctttcacccacctcttctctg |
| *ushA*-U-F | ttaccaaccgaactatcttaaaaaatttca |
| *ushA*-U-R | cgcgcctactgaaagtacc |
| *ushA*-D-F | gcggacatctacttcgcag |
| *ushA*-D-R | tcttgtccttgctcctgatagg |
| *pnnN*-U-F | actaaaaagcttggaaaaaaccagc |
| *pnnN*-U-R | tgctctggtggatcggtc |
| *pnnN*-D-F | cactaaaaatcgagccgcac |
| *pnnN*-D-R | tggttcttgctatgtcagtgg |
| *adeD*-U-F | aaacaaataggggttccgcggaaggtagtgcttggcctt |
| *adeD*-U-R | gctgtgtgtctttgatgacatg |
| *adeD*-D-F | catgtcatcaaagacacacagcgcgccttggtagtaaaggt |
| *adeD*-D-R | tctccttacgcatctgtgcgccttttctatcccaatcctcttc |
| *dns-*sgRNA | ccgctttgccagcattgcagctg |
| *hutH-*sgRNA | ccgcattgttcctgcagcggaag |
| *vpsT-*sgRNA | ccgacttgcagagtttggacgtc |
| *vpsR-*sgRNA | ccgcttggaatctctgctcgttc |
| *pta1-*sgRNA | ccgcgtcgtactgtagaggaccg |
| *pnnN-*sgRNA | ccacccggaaatatcacaatgcc |
| *ScSAM2-*sgRNA | cctttacgccgtgtatacgcccg |


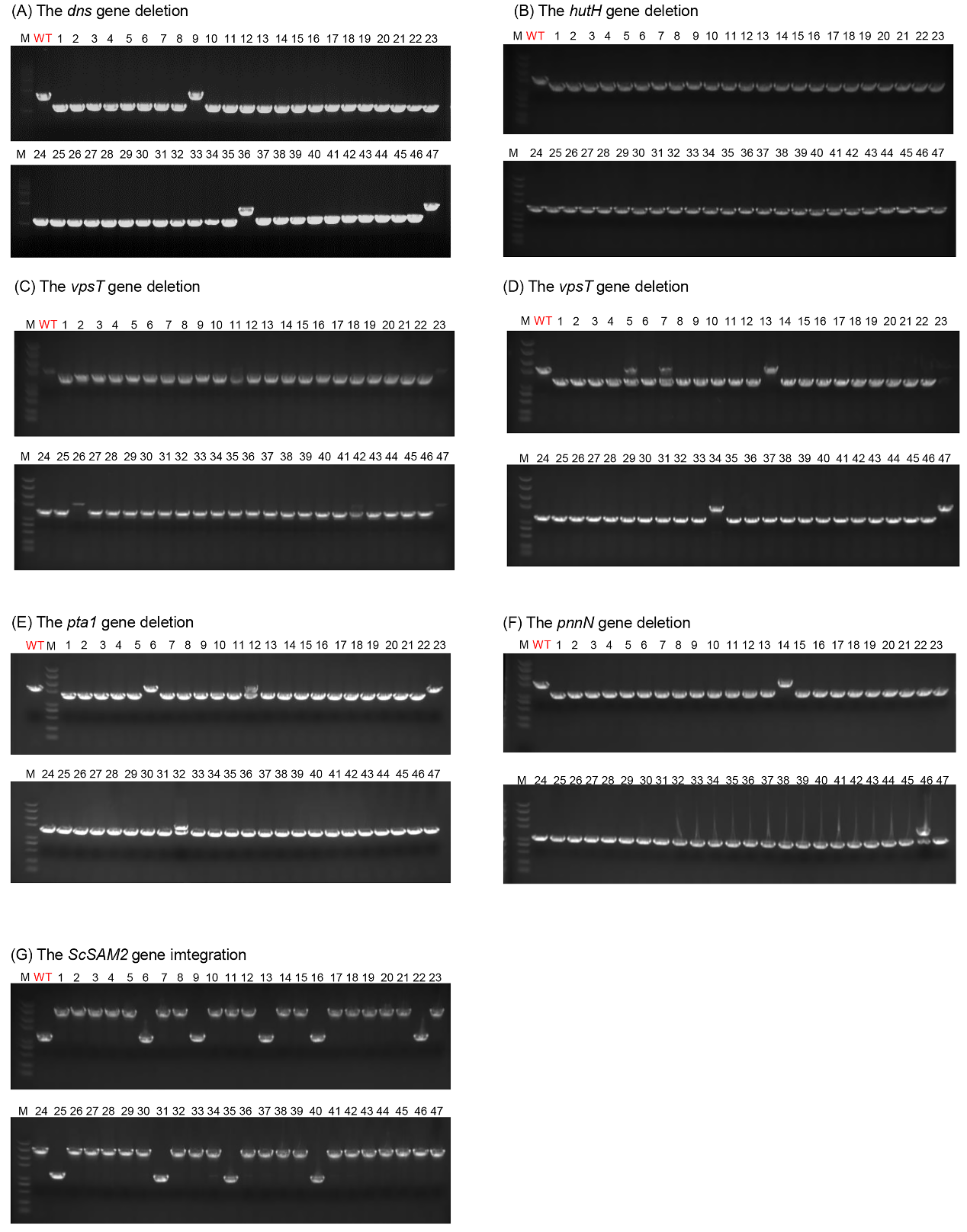


Fig. S1. Agarose gel electrophoresis validation of the consecutive deletion and integration in *V. natriegens* by the dual-plasmid CRISPR-Cas9 editing system. In this study, 47 colonies were analyzed by colony PCR and DNA sequencing, and the desired sequence was identified in all positive clones. Lane 1, marker; Lane 2, wild type; Lanes 3–50, transformants.


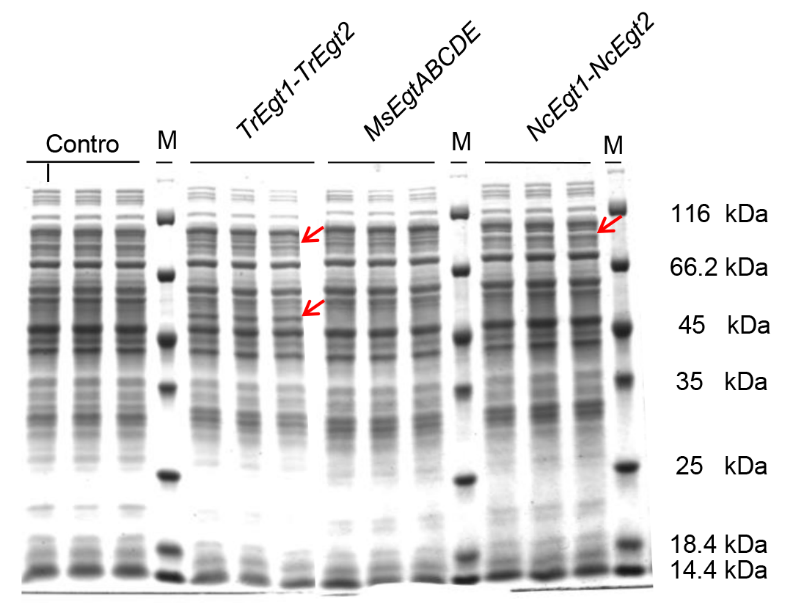


Fig. S2. Analysis of the protein expression profile of the engineered strain V. nEgt02 expressing TrEgt1 and TrEgt2 from *T. reesei*, V. nEgt 03 expressing NcEgt1–NcEgt2 from *N. crassa*, and V. nEgt 04 expressing MsEgtABCDE from *M. smegmatis*.


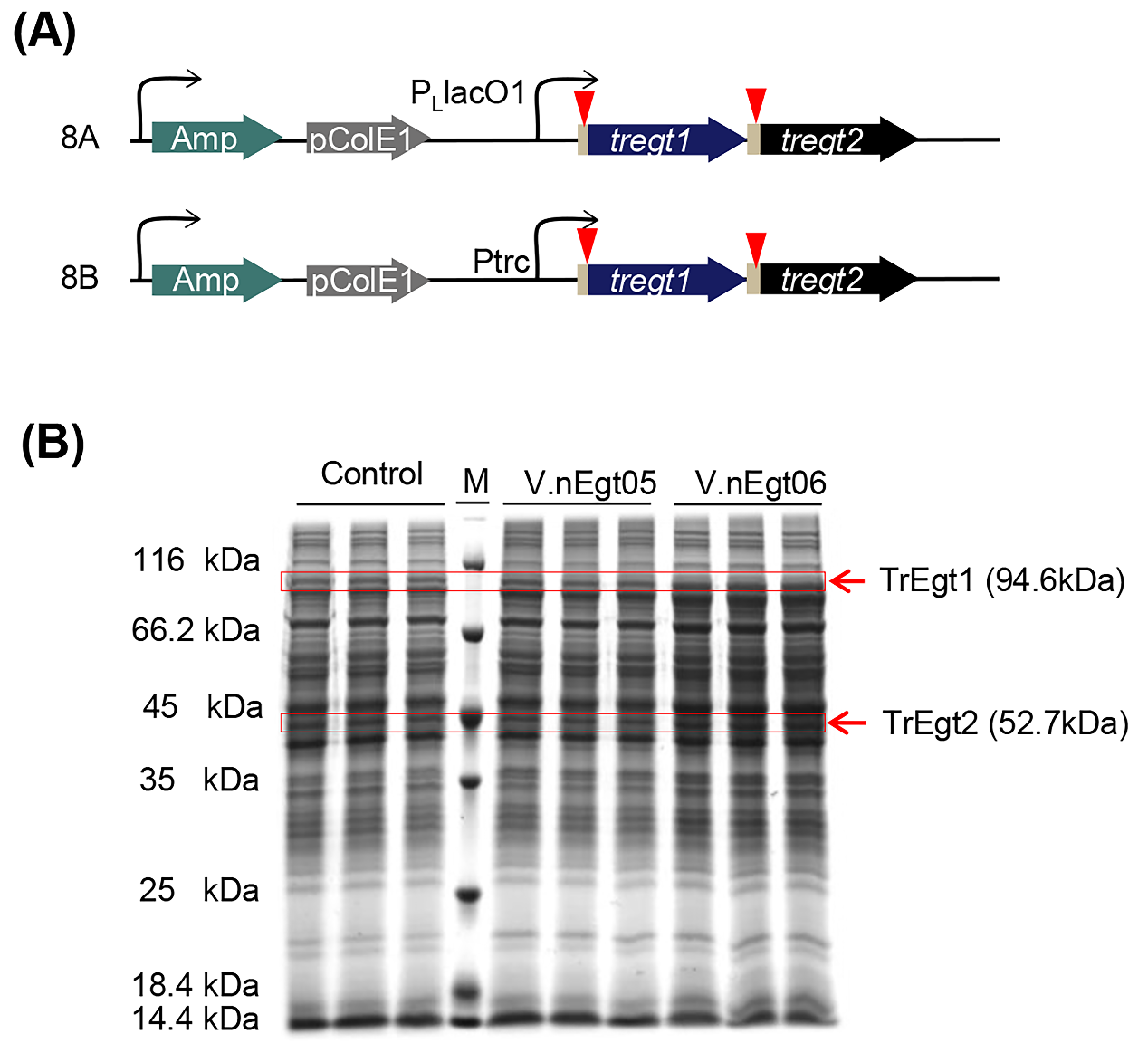


**Fig. S3.** (A) Visualization of recombinant plasmid 8A containing the combination of the IPTG-inducible strong promoter Ptrc and original RBS and plasmid 8B containing the combination of the superstrong P_L_*lacO1* promoter and original RBS, which were used to construct the engineered strains V. nEgt05 and V. nEgt06, respectively. The substitutions of the original RBS with RBS7 are marked by red inverted triangles. (B) Detection of Tregt1 and Tregt2 expression in the engineered strains V. nEgt05 and V. nEgt06 by SDS‒PAGE. Tregt1 and Tregt2 polypeptides are indicated by red arrows. Control, V. nEgt02 containing plasmid 7A with a combination of pBAD and the original RBS. M, protein marker; TrEgt1, 94.6 kDa; TrEgt2, 52.7 kDa.


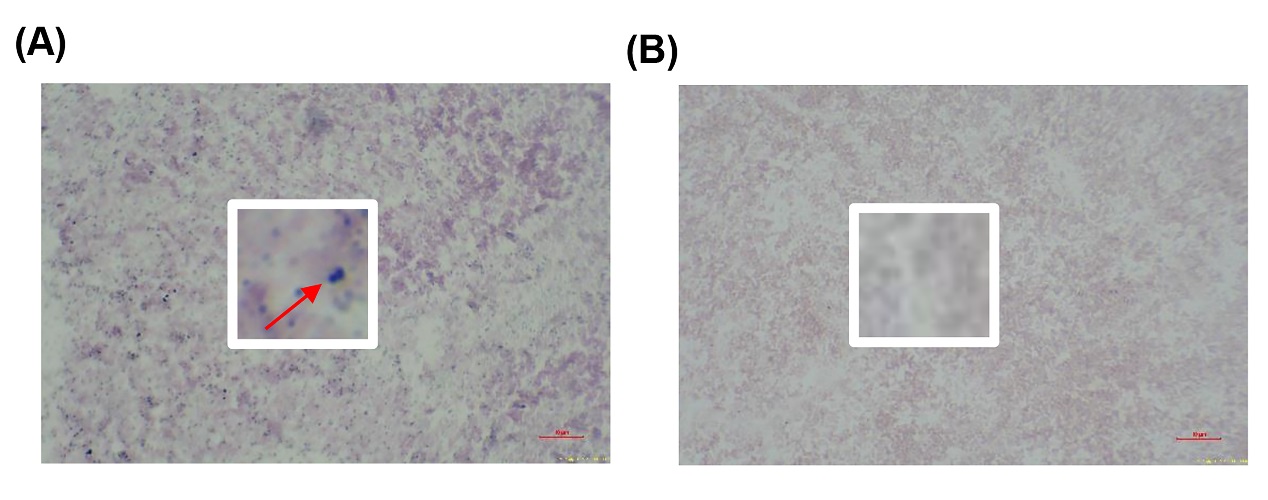


Fig. S4. Electron microscopy images of the *V. natriegens* wild type strain (A) and *phaB* mutant (B) stained with Sudan black B. (*Inset*) The red arrow shows the presence of PHB in the form of black granules inside bacterial cells. Scale bar: 10 µm.


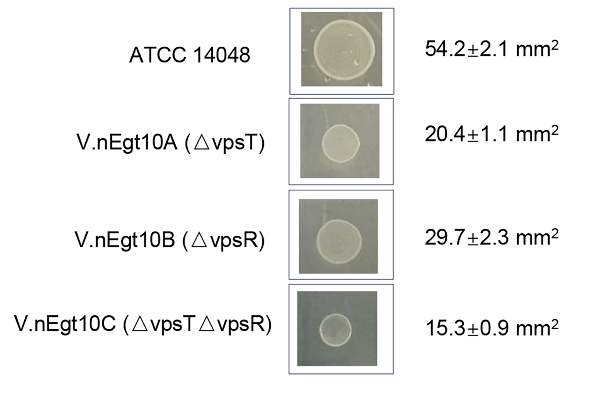


Fig. S5. Representative image of the LB3 solid plate assay of the indicated strains with the quantification of the bacterial colony diameters from six biological replicates shown as the means ± SDs.

**
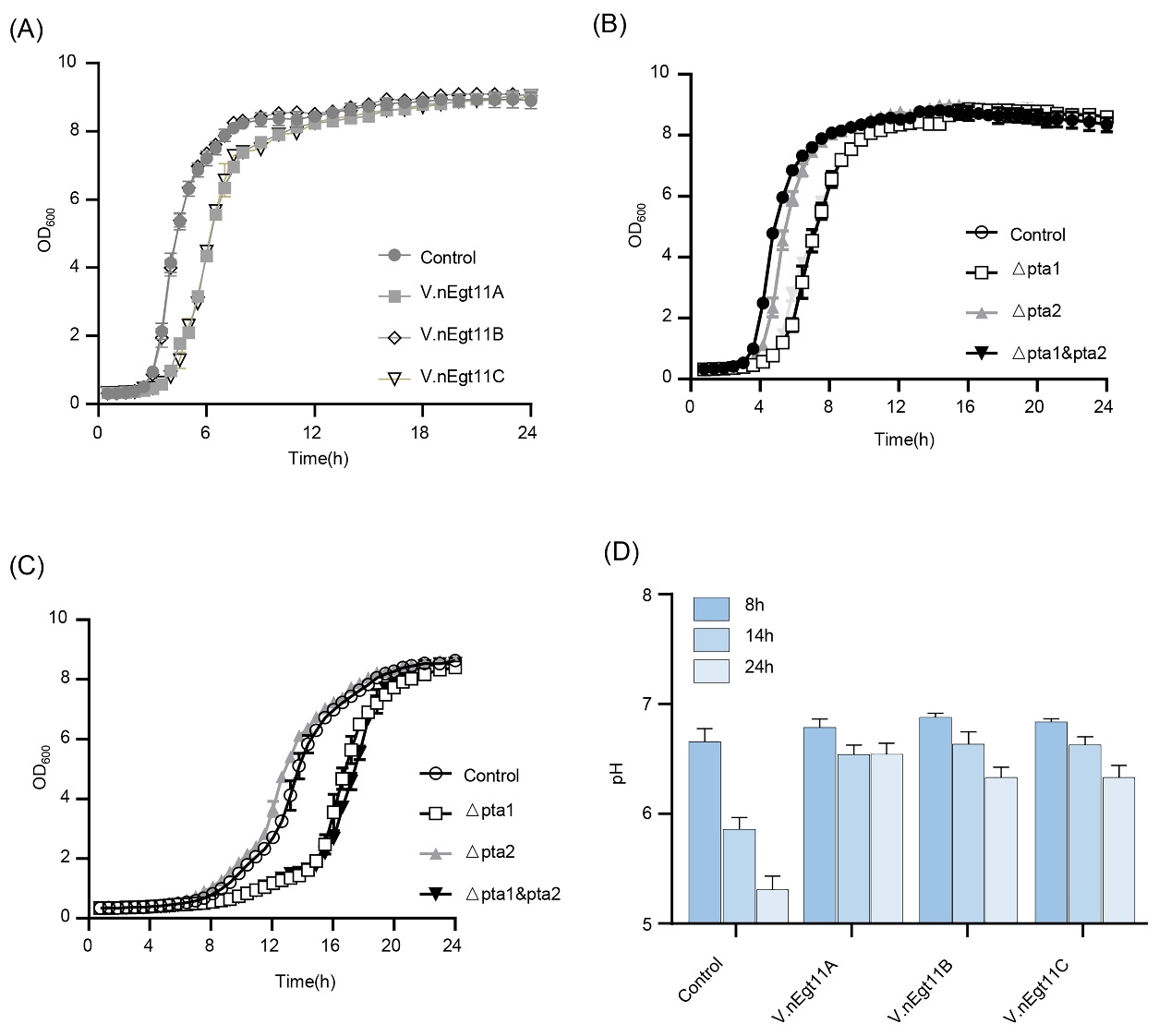
**

Fig. S6. (A) Growth curves of the *pta*-deleted strains V. nEgt11A (△*pta1*), V. nEgt11A (△*pta2*), and V. nEgt11C (△*pta1*△*pta2*) on LB medium. (B) Growth curves of the *pta*-deleted strains at the low concentration of acetate (13 mM) as the sole carbon source. (C) Growth curves of the *pta*-deleted strains at the high concentration of acetate (122mM) as the sole carbon source. (D) Monitoring the pH of the fermentation broth of the *pta*-deleted strains. Control: V. nEgt10C.


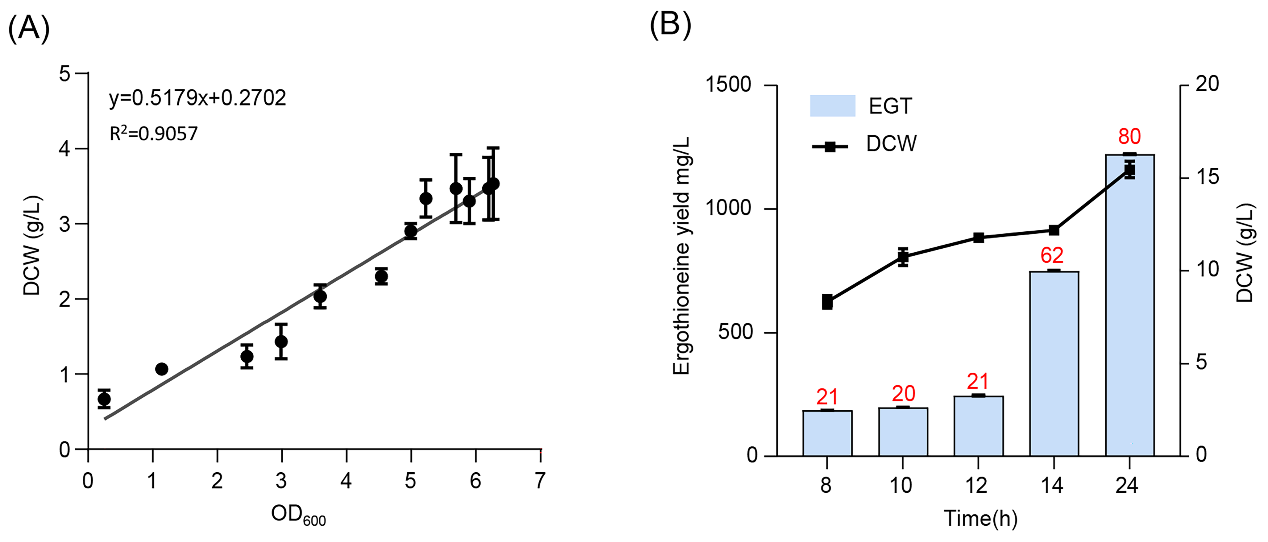


Fig S7 (A) Determining OD_600_ and DCW after 12 h incubation of the engineered strain V. nEgt14AB in shake flasks, indicating a high correlation between OD and DCW. (B) Ergothioneine yield of V. nEgt14AB under non-sterile fed-batch fermentation conditions in a 2-L bioreactor. The numbers in red and above the column chart indicate the content of ergothioneine (mg/g DCW).
